# Cell-Type Specific Roles for PTEN in Establishing a Functional Retinal Architecture

**DOI:** 10.1371/journal.pone.0032795

**Published:** 2012-03-05

**Authors:** Robert Cantrup, Rajiv Dixit, Elena Palmesino, Stephan Bonfield, Tarek Shaker, Nobuhiko Tachibana, Dawn Zinyk, Sarah Dalesman, Kazuhiro Yamakawa, William K. Stell, Rachel O. Wong, Benjamin E. Reese, Artur Kania, Yves Sauvé, Carol Schuurmans

**Affiliations:** 1 Department of Biochemistry and Molecular Biology, Hotchkiss Brain Institute, Alberta Children's Hospital Research Institute, University of Calgary, Calgary, Alberta, Canada; 2 Department of Medicine, Institut de Recherches Cliniques de Montréal (IRCM), Université de Montréal, Montréal, Quebec, Canada; 3 Department of Cell Biology and Anatomy, Hotchkiss Brain Institute, Alberta Children's Hospital Research Institute, University of Calgary, Calgary, Alberta, Canada; 4 Department of Psychological and Brain Sciences, Neuroscience Research Institute, University of California Santa Barbara, Santa Barbara, California, United States of America; 5 Department of Physiology and Pharmacology, Hotchkiss Brain Institute, Alberta Children's Hospital Research Institute, University of Calgary, Calgary, Alberta, Canada; 6 Laboratory of Neurogenetics, RIKEN Brain Science Institute, Wako-shi, Saitama, Japan; 7 Department of Biological Structure, University of Washington, Seattle, Washington, United States of America; 8 Department of Ophthalmology, University of Alberta, Edmonton, Alberta, Canada; Seattle Children's Research Institute, United States of America

## Abstract

**Background:**

The retina has a unique three-dimensional architecture, the precise organization of which allows for complete sampling of the visual field. Along the radial or apicobasal axis, retinal neurons and their dendritic and axonal arbors are segregated into layers, while perpendicular to this axis, in the tangential plane, four of the six neuronal types form patterned cellular arrays, or mosaics. Currently, the molecular cues that control retinal cell positioning are not well-understood, especially those that operate in the tangential plane. Here we investigated the role of the PTEN phosphatase in establishing a functional retinal architecture.

**Methodology/Principal Findings:**

In the developing retina, PTEN was localized preferentially to ganglion, amacrine and horizontal cells, whose somata are distributed in mosaic patterns in the tangential plane. Generation of a retina-specific *Pten* knock-out resulted in retinal ganglion, amacrine and horizontal cell hypertrophy, and expansion of the inner plexiform layer. The spacing of *Pten* mutant mosaic populations was also aberrant, as were the arborization and fasciculation patterns of their processes, displaying cell type-specific defects in the radial and tangential dimensions. Irregular oscillatory potentials were also observed in *Pten* mutant electroretinograms, indicative of asynchronous amacrine cell firing. Furthermore, while *Pten* mutant RGC axons targeted appropriate brain regions, optokinetic spatial acuity was reduced in *Pten* mutant animals. Finally, while some features of the *Pten* mutant retina appeared similar to those reported in *Dscam*-mutant mice, PTEN expression and activity were normal in the absence of *Dscam*.

**Conclusions/Significance:**

We conclude that *Pten* regulates somal positioning and neurite arborization patterns of a subset of retinal cells that form mosaics, likely functioning independently of *Dscam*, at least during the embryonic period. Our findings thus reveal an unexpected level of cellular specificity for the multi-purpose phosphatase, and identify *Pten* as an integral component of a novel cell positioning pathway in the retina.

## Introduction

Patterning of retinal neurons in the radial or vertical dimension allows for the directional flow of visual information. Light first stimulates photoreceptors in the outer nuclear layer (ONL), which then signal through interneurons in the inner nuclear layer (INL); the latter transform visual information and finally relay it to retinal ganglion cells (RGCs) in the ganglion cell layer (GCL), which in turn transmit visual information to the brain. A further refinement of cellular spacing occurs along the tangential (horizontal) plane, with cone photoreceptors, horizontal cells, amacrine cells and RGCs forming non-random cellular arrays or mosaics that evenly tile the retinal field [1]. The processes of retinal neurons also arborize and synapse in precise patterns, sorting into specific sublaminar compartments arranged vertically in the outer (OPL) and inner (IPL) plexiform layers, while in the horizontal plane, retinal neurites disperse in regularly spaced arrays to provide complete visual coverage [2]. Currently, the molecular mechanisms that specifically direct individual types of retinal cells into their proper laminar and mosaic positions, where they establish subtype-specific arborization patterns, are not completely understood.

Each retinal cell type follows distinct migratory routes to reach its final destination. For instance, as retinal progenitor cells differentiate into RGCs, they lose their apical processes and retain their basal contact, which becomes the axon and helps to “pull” RGCs into their laminar position in the GCL [3], [4]. In contrast, amacrine cells lose both apical and basal attachments upon differentiation, and this allows them to migrate more freely into the INL and GCL, likely in response to environmental cues [1]. Globally, the vertical migration of retinal cells depends on the extracellular matrix [5] and the proper establishment of apicobasal cell polarity [1]. In contrast, the molecular regulation of cellular positioning in the tangential plane is less well understood. At the cellular level, retinal mosaics of each cell type develop cell-autonomously, independently of those formed by other cell types [6]. Minimal distance spacing rules are the primary determinant of somal patterning in retinal cell mosaics [7]. However, the cellular mechanisms that establish these minimal distances between homotypic cells are cell type-specific, and can involve negative feedback regulation of cell fate specification, tangential dispersion and/or programmed cell death [8]. Similarly, the rules governing the distribution of retinal cell dendrites in the tangential plane also vary, depending upon the cell type in question [7]. One of the few molecules known to regulate retinal cell spacing and neurite arborization in the tangential plane is DSCAM, a homophilic cell adhesion molecule of the immunoglobulin superfamily (IgSF). DSCAM controls the spacing, as well as the neuritic arborization patterns, of specific RGC and amacrine cell subtypes, disrupting these cellular mosaics and leading to cell and neurite clustering [9]–[13]. Interestingly, in the mammalian retina, DSCAM is thought to control cell spacing and dendritic patterning by blocking responsiveness to unknown adhesive signals, resulting in a “gain” of adhesiveness in *Dscam* mutant retinas that leads to the specific clumping of amacrine and RGC somata and processes in ectopic locales along the radial and tangential dimensions. Currently, the identities of the adhesive signals that are blocked by DSCAM to regulate radial/tangential dispersion are unknown. However, several other cell adhesion molecules have been shown to pattern the stratification of retinal cell neurites/processes in the vertical axis of the IPL; these include the related IgSF molecules DscamL and Sidekick1/2 [14], the atypical cadherin Fat3 [15], as well as class 5 and 6 semaphorins and their plexin receptors [16].

*Pten* (*phosphatase and tensin homolog*) encodes a lipid and protein phosphatase that negatively regulates phosphoinositide-3-kinase (PI3K) signalling and controls cell growth and migration in multiple tissues [17]–[21]. In the nervous system, *Pten* mutations lead to neuronal hypertrophy and defects in cell migration, dendrite arborization and myelination [17], [18], [22]–[26]. In the retina, the ectopic activation of PI3K signaling results in defective cell migration [27]. Similarly, a recent report has demonstrated that the retina-specific deletion of *Pten*, which results in elevated PI3K signalling, results in deficits in retinal cell differentiation and migration and abnormal physiological responses of the mutant retina to light [28]. Here we used a different retina-specific cre driver, identifying several additional functions for *Pten* in retinal development, while corroborating some of the previously reported findings [28]. Specifically, we report that PTEN is preferentially expressed in subpopulations of mosaically-patterned retinal cells, all of which become hypertrophic in retina-specific *Pten* conditional knock-outs (cKO). We also identify specific defects in cellular patterning and neurite arborization in *Pten* mutant retinal ganglion, horizontal and amacrine cells. These *Pten* mutant cellular defects appear to result in abnormalities of visual processing, as revealed by deficits in ERG recordings and optokinetic responses. Finally, although the retinal phenotypes in *Pten* cKO and *Dscam* KO show some similarities, we find that PTEN expression and activity are not altered in *Dscam* mutants. *Pten* is thus a novel component of the cellular growth, positioning and neurite arborisation pathway(s) that operate within retinal cells that form non-random cellular arrays or mosaics.

## Results

### Generation of retinal-specific *Pten* cKOs

**Figure 1:**
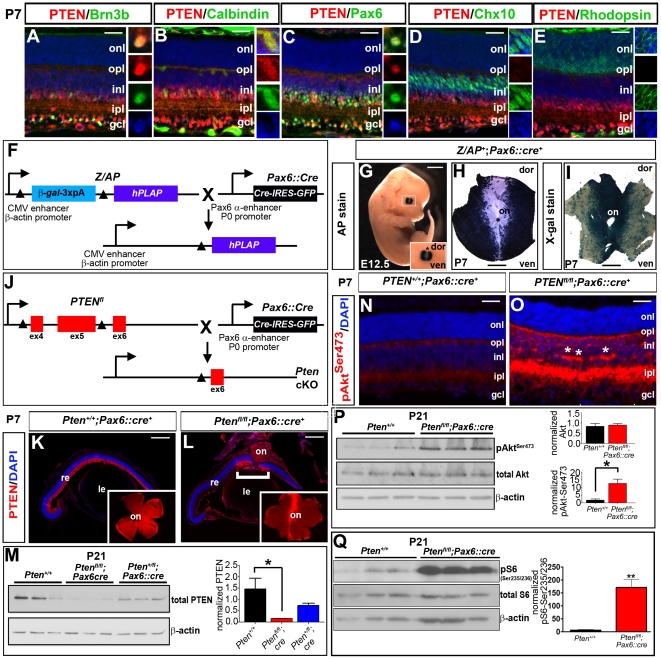
PTEN retinal expression and generation of retinal-specific *Pten* cKO. (A–E) Co-labeling of P7 retina with PTEN (red) and Brn3b (green, A), calbindin (green, B), Pax6 (green, C), Chx10 (green, D) and rhodopsin (green, E). Blue is DAPI counterstain. Insets to the right of each panel are high magnification images of PTEN^+^ cells, showing co-expression in Brn3b^+^ RGCs (A), calbindin^+^ horizontal cells (B) and Pax6^+^ amacrine cells (C). Insets in D show low levels of PTEN co-expression in Chx10^+^ bipolar cells, while PTEN protein was not detected in rhodopsin^+^ rod photoreceptors (E). (F–I) Schematic illustration of crosses between transgenic animals carrying a Z/AP dual reporter and *Pax6* α-cre/P0 promoter::*Cre-IRES-GFP* transgene (hereafter designated *Pax6::Cre*) (F). Analysis of AP histochemical stain (i.e., recombined cells) in *Pax6::Cre*;Z/AP double transgenics at E12.5 (G) and in a P7 retinal flatmount (H). β-galactosidase histochemical stain (i.e., non-recombined cells) in a P7 retinal flatmount (I). Inset in G is a high magnification image of the eye, with the asterisk designating a lack of recombination in the central retina. (J) Schematic illustration of crosses between mice carrying a floxed *PTEN* allele (*PTEN^fl^*) and *Pax6::Cre* transgene. (K–M) Expression of PTEN in P7 *Pten* ^+/+^ (K) and *Pten* cKO (L) retinal sections. Bracket in L shows central retina where *Pten* is not deleted and expression is maintained. Insets in K and L show PTEN immunolabeling of retinal flatmounts, confirming that PTEN expression is retained in the central retina in *Pten* CKOs. PTEN Western blot analysis and densitometry on P21 wild-type and *Pten* heterozygous and homozygous cKO retinae (M). (N,O) Expression of pAkt^Ser473^ in P7 wild-type (N) and *Pten* cKO (O) retinae. Asterisks in O mark aberrant aggregations of amacrine dendrites in the INL. (P,Q) Western blot analysis and densitometry of pAkt^Ser473^/Akt (P) and pS6^Ser235/236^/S6 (Q) in P21 wild-type and *Pten* cKO retinal lysates. p values are denoted as follows: <0.05 *, <0.01 **, <0.005 ***. gcl, ganglion cell layer; inl, inner nuclear layer; ipl, inner plexiform layer; le, lens; on, optic nerve; onbl, outer neuroblast layer; onl, outer nuclear layer; opl, outer plexiform layer; re, retina. Scale bars = 50 µm (A–E,N,O), 2 mm (G), 1 mm (H,I), 600 µm (K,L).

We reasoned that the molecules involved in regulating cell type-specific migration patterns in the retina would be expressed in distinct sets of actively migrating, newly differentiated retinal cells. By screening the expression profiles of several signal transduction molecules in embryonic and early postnatal mouse retinas, we revealed that several components of the PI3K signalling pathway – including the PTEN phosphatase – were expressed in a restricted manner in differentiating retinal neurons. From embryonic day (E) 12.5 to P0, PTEN protein was detected at low levels in the outer neuroblastic layer (onbl), where proliferating progenitors reside, and at higher levels in the developing INL and GCL (data not shown). By postnatal day (P)7, when retinal differentiation is complete except in the peripheral-most retina, PTEN expression was detected in a subset of postmitotic retinal cells, including Brn3b^+^ RGCs, calbindin^+^ horizontal cells, and Pax6^+^ amacrine cells and RGCs (Figure 1A–C). In contrast, PTEN immunoreactivity was low in Chx10^+^ bipolar cells and undetectable in rhodopsin^+^ rod photoreceptors (Figure 1D,E). Furthermore, in the plexiform layers, PTEN was co-localized in syntaxin^+^ amacrine cell processes, SMI-32^+^ RGC dendrites, and calbindin^+^ horizontal cell processes (Figure 1B; data not shown). During retinal development, PTEN is thus preferentially expressed in the cell bodies and processes of retinal neurons that form mosaics, including RGCs, amacrine and horizontal cells.

Given that *Pten* regulates cell size, migration and neurite arborization in several CNS domains [17], [24]–[26], we predicted that it may play a critical role in retinal development. To analyze the requirement for *Pten* in the developing retina, we used a conditional loss-of-function approach, taking advantage of a retina-specific *Cre* transgene (*Pax6* α-enhancer/P0 promoter::*Cre-IRES-GFP*; hereafter named *Pax6::cre*) that promotes recombination in the peripheral retina beginning at E10.5, targeting progenitors for all retinal cell types [29]. *Pax6::cre* transgenics were crossed with mice carrying a Z/AP dual reporter (Figure 1F) [30]. To confirm that cre-mediated excision occurred, a histochemical stain for alkaline phosphatase (AP), which is transcribed upon cre-mediated excision, was performed, and shown to specifically label cells in the peripheral retina of Z/AP;*Pax6::cre* transgenics at E12.5 and in P7 flatmounts (Figure 1G,H). Conversely, β-galactosidase activity (marking non-recombined cells) was more intense in the central retina (Figure 1I). *Pax6::cre* mice were then crossed with animals carrying a floxed *Pten* allele (floxed exons 4/5; designated *Pten* ^fl^; Figure 1J) [18]. As expected from our reporter analyses, PTEN immunolabeling was reduced, except in the central-most retina in P7 *Pten* ^fl/fl^;*Pax6::cre* animals (hereafter designated *Pten* cKO; Figure 1L *vs* 1K); this reduction was confirmed by Western blotting at P21 (Figure 1M).

PTEN is a negative regulator of PI3K, which phosphorylates and activates membrane inositol phospholipids. pAkt^Ser473^ is a readout of active PI3K signalling, the phosphorylation of which is reversed by the PTEN phosphatase. Accordingly, pAkt^Ser473^ levels were elevated in the peripheral GCL, INL, IPL and OPL in P7 *Pten* cKO retinas (Figure 1O *vs* 1N), and the increase in pAkt^Ser473^ levels was confirmed by Western blotting of P21 retinal lysates (Figure 1P). Furthermore, phosphorylation of S6 (pS6^Ser235/236^), a downstream effector of mTOR signalling that is also regulated by the PI3K pathway, was also increased (21-fold) in P21 *Pten* cKO retinas (Figure 1Q). Thus, PI3K and mTOR signalling, which are negatively regulated by PTEN, are strikingly upregulated as a consequence of deleting *Pten* in the retina.

### *Pten* is required to establish a normal retinal architecture and regulate cell size

**Figure 2:**
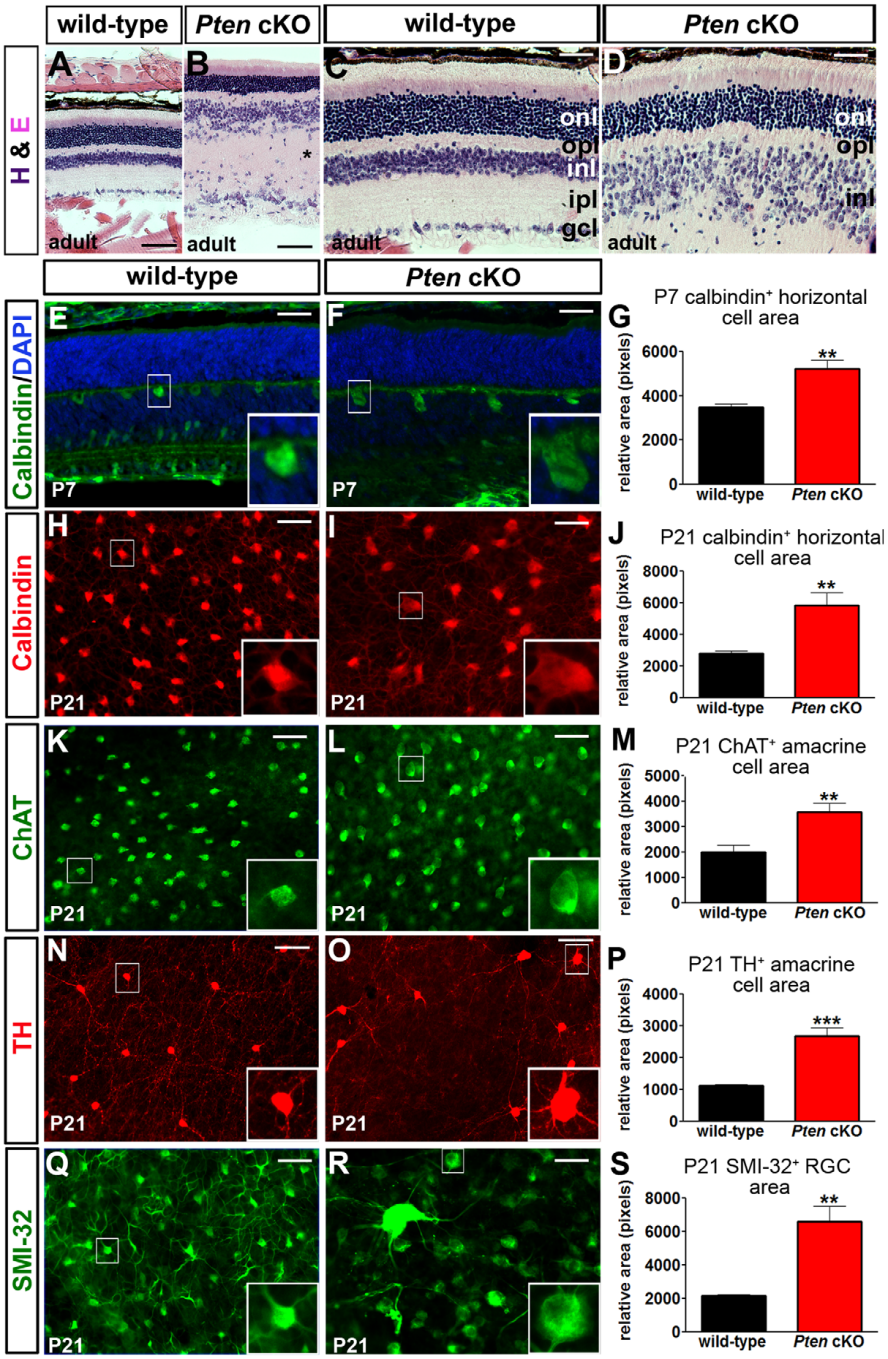
Abnormal retinal architecture and increased retinal cell sizes in *Pten* cKOs. (A–D) Low (A,B) and high (C,D) magnification images of hematoxylin-eosin (H&E) stained sections of adult wild-type (A,C) and *Pten* cKO (B,D) retinae. (E–G) Calbindin labelling of P7 wild-type and *Pten* cKO retinal sections (E,F) and area measurements of calbindin^+^ horizontal cells (G). (H–S) Labeling of retinal flatmounts from P21 wild-type and *Pten* cKOs with calbindin (H,I), ChAT (K,L), TH (N,O), and SMI32 (Q,R). Calculation of cell areas for P21 calbindin^+^ horizontal cells (J), ChAT^+^ (M) and calbindin^+^ (P) amacrine cells and SMI32^+^ RGCs (S). p values are denoted as follows: <0.05 *, <0.01 **, <0.005 ***. Scale bars = 100 µm (A,B,N,O), 50 µm (C–L,Q,R).

To determine whether *Pten* was globally required for retinal morphogenesis, histological sections of adult wild-type and *Pten* cKO retinas were analyzed. While the three cellular and two plexiform layers were readily visible in both wild-type and *Pten* mutant retinas (Figure 2A–D), several abnormalities were apparent in *Pten* cKOs, including: 1) a striking increase in retinal thickness; 2) a grossly expanded IPL populated by ectopic cells; 3) an expanded, loosely packed INL in which nuclei appeared larger, and 4) a thinner ONL. These results suggested that *Pten* may influence several events during retinal development, including cellular differentiation, cell migration, and neurite outgrowth and arborization, each of which was then individually investigated.

*Pten* mutations are associated with cellular hypertrophy in several tissues, largely because of the role that PTEN plays in negatively regulating mTOR-p70S6K-S6 signalling, a key cell growth pathway [18], [22]–[24], [31]. Given that pS6 levels were elevated in *Pten* cKO retinas at P21 (Figure 1Q), we investigated whether the loss of *Pten* resulted in an increase in retinal cell sizes (Figure 2). We found that the cross-sectional areas of calbindin^+^ horizontal cell bodies in *Pten* cKO retinas were 1.5 times the area of wild-type horizontal cells at P7 (Figure 2E–G; Table S1), increasing to 2.1 times the normal area at P21 (Figure 2H–J; Table S1). Furthermore, in P21 retinal flatmounts, the cross-sectional areas of choline acetyltransferase-positive (ChAT^+^; 2.1-fold increase; Figure 2K–M; Table S1) and tyrosine hydroxylase-positive (TH^+^; 2.4-fold increase; Figure 2N–P; Table S1) amacrine cell somata were significantly larger in *Pten* cKO retinas compared to wild-type retinas, as were SMI-32^+^ RGCs (3.1 times normal; Figure 2Q–S, Table S1).

Thus, the sizes of somata of the three retinal cell types that express high levels of PTEN are increased in *Pten* cKOs, as expected from the observed increase in mTOR signalling in these mutant retinas.

### *Pten* regulates the positioning of RGCs, amacrine and horizontal cell bodies

A fundamental feature of the mature retina is that neuronal cell bodies are positioned in discrete strata within a nuclear layer for a given type of cell, and that cells of a given type are positioned at regular intervals across that stratum, facilitating complete and economical sampling of the visual field [32]. These two features of a functional retinal architecture are produced during development through the radial and tangential dispersion of newborn neuroblasts [33]. Our histological analyses provided evidence for aberrant radial migration in the absence of *Pten* function, as ectopic cells were detected in the *Pten* cKO IPL (Figure 2B,D). Furthermore, Pax6^+^ amacrine cells and Brn3a^+^ RGCs were detected in the *Pten* cKO IPL, indicating that subsets of amacrine cells and RGCs migrate aberrantly along the radial axis (Figure S1).

**Figure 3:**
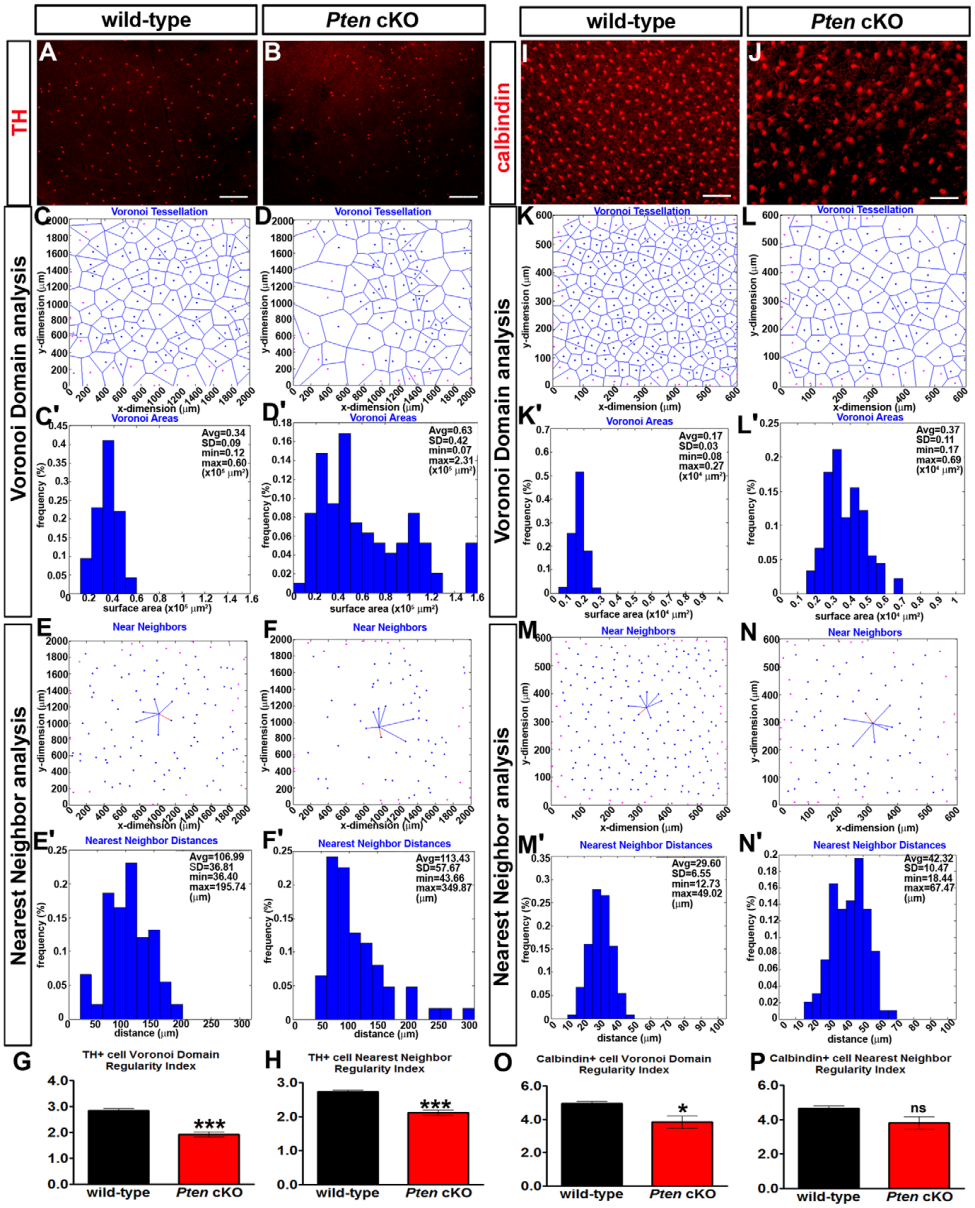
Aberrant cellular mosaicism in *Pten* cKOs. (A–H) Immunolabeling of P21 wild-type (A) and *Pten* cKO (B) retinal flatmounts with TH. Voronoi diagrams depicting the distribution of TH^+^ amacrine cells in P21 wild-type (C) and *Pten* cKO (D) retinae. Calculation of TH^+^ Voronoi domain areas and their relative distributions in these two fields for P21 wild-type (C′) and *Pten* cKO (D′) retinae. Near neighbors of a TH^+^ reference cell in P21 wild-type (E) and *Pten* cKO (F) retinae, with the nearest neighbour indicated in red. Frequency distribution of nearest neighbor distances between TH^+^ amacrine cells in these two fields for P21 wild-type (E′) and *Pten* cKO (F′) retinae. Calculation of Voronoi domain (G) and Nearest Neighbor (H) regularity indices for TH^+^ amacrine cells in wild-type and *Pten* cKO retinae. (I–P) Immunolabeling of P21 wild-type (I) and *Pten* cKO (J) retinal flatmounts with calbindin. Voronoi diagrams depicting the distribution of calbindin^+^ horizontal cells in P21 wild-type (K) and *Pten* cKO (L) retinae. Calculation of TH^+^ Voronoi domain areas and their frequency distributions in P21 wild-type (K′) and *Pten* cKO (L′) retinae in these two fields. Near neighbors of a calbindin^+^ reference cell in P21 wild-type (M) and *Pten* cKO (N) retinae, with the nearest neighbour indicated in red. Frequency distribution of distances between TH^+^ amacrine cells in P21 wild-type (M′) and *Pten* cKO (N′) retinae in these two fields. Calculation of Voronoi domain (O) and Nearest Neighbor (P) regularity indices for calbindin^+^ horizontal cells in wild-type and *Pten* cKO retinae. p values are denoted as follows: <0.05 *, <0.01 **, <0.005 ***. Scale bars = 600 µm (A,B), 100 µm (C,D).

To test whether *Pten* also regulates the tangential dispersion of retinal cells, we assessed the mosaic distribution of horizontal and amacrine cell subtypes in P21 retinal flatmounts. Anti-TH was used to label dopaminergic amacrine cells. In wild-type retinae, TH^+^ cell somata mosaics were dispersed in a patterned array (Figure 3A) [34]. In contrast, in *Pten* cKO retinas, TH^+^ amacrine cells appeared less regularly distributed (Figure 3B). To quantify the regularity of the cellular spacing in these mosaics, we examined their spatial properties using Voronoi domain and nearest neighbor analyses. Voronoi domain analysis computes the territory surrounding each cell that is closer to that cell than any of the neighboring cells [35]. Visual analysis of these Voronoi diagrams (Figure 3C,D) and of plots comparing the frequency of domain areas (Figure 3C′,D′) revealed a greater variability in domain sizes in *Pten* cKO retinae. To analyze this quantitatively, the Voronoi domain regularity index was calculated for each individual field (average domain area/standard deviation; Figure 3G), with higher values indicative of more regular spacing. As expected, TH^+^ regularity indices derived from this Voronoi tessellation were significantly lower in *Pten* cKO retinae (p<0.0001; Figure 3G, Table S2). Nearest neighbour analyses revealed a comparable increase in the variability of this measure (Figure 3E,F), as revealed by the skewed distribution of nearest neighbour distances for the TH^+^ amacrine cells in *Pten* cKO retina (Figure 3E′F′), resulting in a significant decrease in the nearest neighbor regularity index (p<0.0001; Figure 3H).

We used similar analyses to examine the distribution of calbindin^+^ horizontal cells in P21 wild-type and *Pten* cKO retinal flatmounts (Figure 3I,J). By generating the Voronoi tessellation for calbindin^+^ horizontal cells (Figure 3K,L), and by plotting domain sizes (Figure 3K′,L′), it was apparent that calbindin^+^ domains were more irregular in *Pten* cKO retinae. Accordingly, a significant decrease in the Voronoi domain regularity index was observed in *Pten* cKO retinae (p = 0.03; Figure 3O). In contrast, while there was a trend towards more variable calbindin^+^ nearest neighbours in *Pten* cKO retinae (Figure 3 N,N′,O,O′), this difference did not reach statistical signficance (P>0.05; Figure 3P).

*Pten* thus plays a role in the establishment of the mosaic patterns of dopaminergic amacrine cells and calbindin^+^ horizontal cells.

### Amacrine cell and horizontal cell differentiation are perturbed in *Pten* cKO retinae

**Figure 4:**
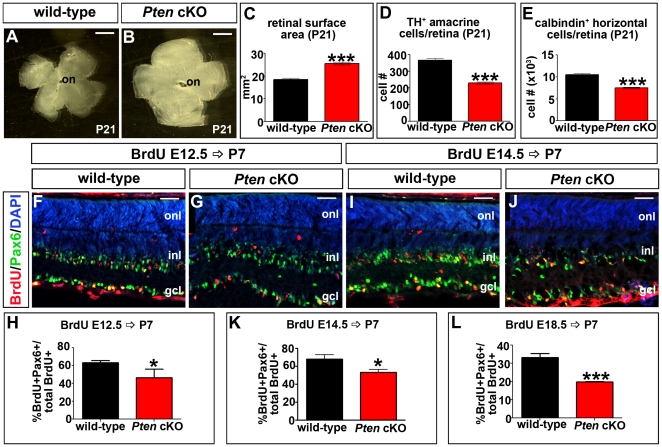
Alterations of retinal surface area and horizontal and amacrine cell numbers in *Pten* cKO retinas. (A–C) Flatmounts of P21 wild-type (A) and *Pten* cKO (B) retinas and calculation of their surface areas (C). (D,E) Quantitation of total numbers of TH^+^ amacrine cells (D) and calbindin^+^ horizontal cells (E) in P21 wild-type and *Pten* cKO retinas. (F–L) Birthdating experiments, with BrdU injected at E12.5 (F–H), E14.5 (I–K) and E18.5 (L) with retinas harvested at P7. Retinas were labeled with Pax6 (green), BrdU (red) and DAPI (blue in F,G,I, J). Quantitation of Pax6^+^BrdU^+^/total BrdU^+^ cells is shown in H, K and L. p values are denoted as follows: <0.05 *, <0.01 **, <0.005 ***. Scale bars = 1 mm (A,B), 50 µm (F,G,I,J).

There are several variables that may negatively influence the regularity of TH^+^ amacrine cell and calbindin^+^ horizontal cell mosaics in *Pten* cKO retinae, including alterations in retinal surface area, cell density, cell death, cell dispersion and cell fate specification [8]. To better understand the disruption of these mosaics in *Pten* cKO, we examined these parameters in more detail. We first measured the overall surface area of the P21 retina, revealing that *Pten* cKO retinae were 1.4-fold larger than wild-type controls (P<0.0001; Figure 4A–C). We then quantitated the density of TH^+^ amacrine cells and calbindin^+^ horizontal cells, and by multiplying this number by the retinal surface area, generated an estimate of total numbers of these cells in individual retinas. Strikingly, both TH^+^ amacrine cells (P<0.0001; Figure 4D) and calbindin^+^ horizontal cells (P<0.0001; Figure 4E) were reduced in number in *Pten* cKO retinas (Table S2). Taken together these data suggested that the loss of *Pten* leads to retinal hypetrophy at the tissue level, but suggest that this is not due to a global increase in cell number; rather, certain populations of retinal cells are present in lower numbers in *Pten* cKO retinas.

We next addressed the mechanisms that might account for the reduction in retinal cell numbers in *Pten* cKO retinae. Apoptosis has been implicated in controlling the tangential distribution of dopaminergic amacrine cells [34], although it is thought to play a lesser role in regulating the formation of horizontal cell mosaics [7]. To visualize apoptotic cells, we monitored the expression of activated caspase-3 (ac-3) in wild-type and *Pten* cKO retinae at E15.5, P0 and P7. The number of ac-3^+^ cells was not noticeably elevated in *Pten* cKO retinas at embryonic and postnatal stages (Figure S2). While the small number of ac-3^+^ cells made it difficult to make a definitive statement, we nevertheless speculated that it may instead be that fewer amacrine and horizontal cells were born in *Pten* cKO retinas during development, rather than these cells undergoing excessive cell death after their birth. To test this, we used birthdating experiments, examining amacrine cell differentiation between E12.5 to E18.5, when most of these cells are born [36]. Given the sparse nature of horizontal cells, similar studies were not performed with this cell type. Bromodeoxyuridine (BrdU) was injected into timed pregnant females at E12.5, E15.5 and E18.5 and retinas were then harvested at P7. By using Pax6 as a pan-amacrine cell marker and quantitating BrdU^+^Pax6^+^/total BrdU^+^ cells, we were able to show that there was a significant decrease in the number of amacrine cells born at E12.5 (P = 0.02; Figure 4F–H), E15.5 (P = 0.04; Figure 4I–K) and E18.5 (P = 0.0007; data not shown) in *Pten* cKO retinas.

There is thus a reduction in amacrine cell differentiation in *Pten* cKO retinas throughout the period of genesis of this cell type. These fewer amacrine cells then become aberrantly distributed in the larger retinal surface area that is characteristic of *Pten* cKO retinas.

### Aberrant IPL sublaminar organization in *Pten* cKOs

**Figure 5:**
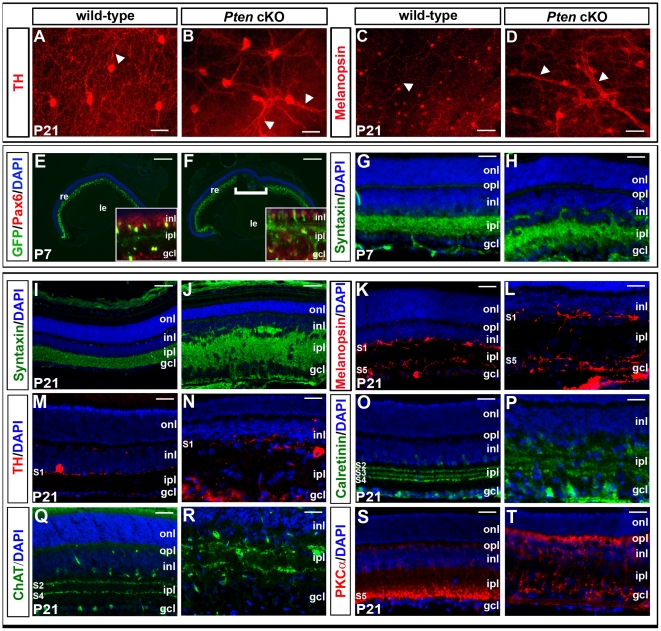
Abnormal patterning of the hypertrophic IPL in *Pten* cKOs. (A–D) P21 wild-type and *Pten* cKO retinae labelled in wholemount for TH (A,B) and melanopsin (C,D). Arrowheads in B,D mark increased fasciculation of TH^+^ amacrine cell processes and melanopsin^+^ RGC dendrites in *Pten* cKO retinae, respectively. (E–H) P7 wild-type and *Pten* cKO retinae labelled with GFP (green; from *Pax6::cre* transgene) and Pax6 (red; E,F) or syntaxin (G,H). Bracket in F marks area where *Pax6::cre* transgene is not expressed. (I–T) P21 wild-type and *Pten* cKO retinae labelled with syntaxin (I,J), melanopsin (K,L), TH (M,N), calretinin (O,P), ChAT (Q,R) and PKCα (S,T). gcl, ganglion cell layer; inl, inner nuclear layer; ipl, inner plexiform layer; le, lens; onl, outer nuclear layer; opl, outer plexiform layer; re, retina. Scale bars = 50 µm (A,B,G,H,K–T), 100 µm (C,D,I,J), 600 µm (E,F).

Because it has been shown that irregularity of cell spacing can influence neurite arborization patterns (and vice versa) [10], we examined the distribution of amacrine cell and RGC processes in the tangential plane of *Pten* cKO retinas. In TH-labeled retinal flatmounts, ectopic fasciculation and bundling of TH^+^ processes was observed in P21 *Pten* cKO retinas, a feature not present in the wild-type retinas (Figure 5A,B). Similarly, the dendrites of melanopsin^+^ ipRGCs were aberrantly bundled in P21 *Pten* cKO retinal flatmounts (Figure 5C,D). *Pten* thus plays a role in uniformly distributing the processes of these two cell types across the retinal surface. In the absence of this phosphatase, RGC dendrites and TH^+^ amacrine cell processes appear to show a preference for adhering to like-type processes, thereby fasciculating abnormally.

The gross expansion of the IPL in *Pten* mutant retinas suggested that patterns of neurite stratification along the radial axis might also be perturbed. Across this dimension, the IPL is comprised of columnar modules of amacrine cell processes, bipolar cell axons and RGC dendrites, which arborize and synapse in discrete layers in a stereotypic fashion. The IPL is subdivided into two broad ON and OFF layers, in which the processes of neurons depolarize or hyperpolarize, respectively, in response to light. Within those two layers, the IPL is further subdivided into discrete strata containing the processes of particular types of bipolar, amacrine and ganglion cells. *Pten* plays a role in the establishment of this stratified architecture of the IPL. A disorganized and expanded IPL was observed as early as P7 in *Pten* cKOs, as revealed by the GFP reporter from the *Pax6::cre* transgene, which was co-localized with Pax6^+^ (in somata of amacrine cells and RGCs) and syntaxin (in amacrine cell processes; Figure 5E–H). By P21, an increasingly expanded and fractionated IPL was present in *Pten* cKOs, as revealed by labelling with pAkt^Ser473^ (Figure 1N,O), syntaxin (Figure 5I,J), and phalloidin (labels actin cytoskeleton; data not shown), in all IPL strata. To assess IPL patterning in more detail, sublamina-specific markers were used. Given the aberrant fasciculation of melanopsin^+^ ipRGC dendrites in the tangential plane, we first examined if these dendrites were similarly perturbed in the radial axis. Melanopsin is expressed in dendrites of type 1 (monostratified, targeting stratum 1) and type 2 (highly branched, targeting inner or proximal IPL) intrinsically photoreceptive RGCs (ipRGCs), and this stratification pattern was normal in *Pten* cKOs (Figure 5K,L). Similarly, the processes of TH^+^ amacrine cells, which were also more heavily fasciculated in the tangential plane, properly targeted substratum 1 in *Pten* cKOs (Figure 5M,N). This was in contrast to amacrine cell processes labelled with calretinin and calbindin, which arborized in three strata in the IPL; and ChAT, a marker for cholinergic starburst amacrine cells, which arborized in OFF (layer 2) or ON (layer 4) sublaminae; all of these arborization patterns were disrupted in P21 *Pten* cKO retinas (Figure 5O–R; 2E,F). Finally, the normal termination zone of rod bipolar cell terminals in sublamina 5, as revealed by labelling for protein kinase C α (PKCα), was severely perturbed in *Pten* cKO retinas, with axonal boutons not restricted to sublamina 5 (Figure 5S,T).

**Figure 6:**
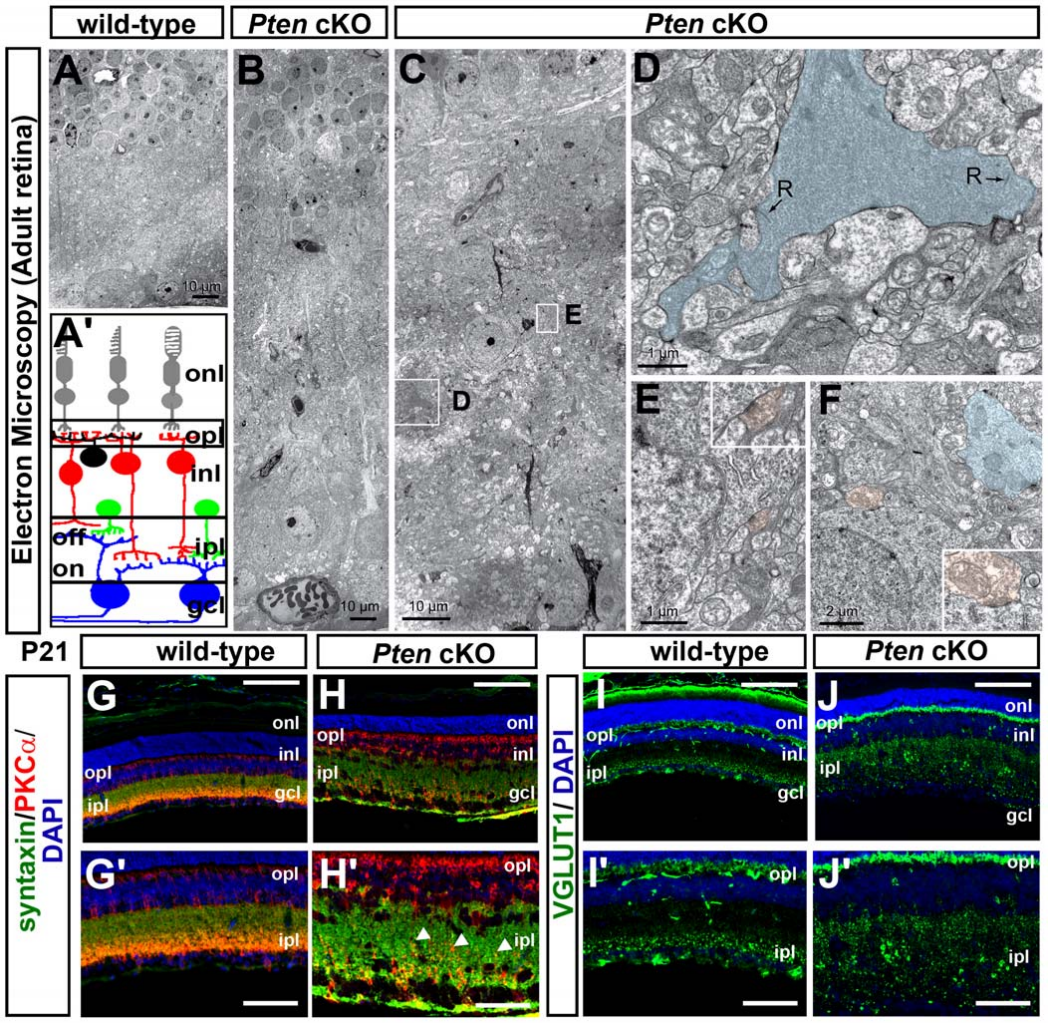
Synaptic contacts in the *Pten* cKO retinal IPL. (A–F) Electron microscopy (EM) of adult wild-type and *Pten* cKO retinae. Schematic illustration of retinal architecture (A′). Low magnification EM images of wild-type (A) and *Pten* cKO (B) retinae, shown to scale, illustrating expansion of mutant retinae. Higher magnification images of *Pten* cKO IPL (C–F), with boxed areas in C shown in higher magnification in D,E. Asterisks in C mark ectopic cells in the IPL. Color scheme in D–F′ is as follows: Blue denotes rod bipolar cell terminal with ribbons (labeled R) in the *Pten* cKO IPL (D). Pink denotes amacrine cell synapses on ectopic somata within the IPL (E,F). GCL, ganglion cell layer; inl, inner nuclear layer; ipl, inner plexiform layer; onl, outer nuclear layer; opl, outer plexiform layer. Scale bars = 10 µm (A,B,C), 1 µm (D,E), 2 µm (F), 100 µm (G–J), 50 µm (G′–J′).

IPL patterning was thus found to be strikingly perturbed in *Pten* cKOs, with defects most evident for amacrine cell processes and bipolar cells. Given that synaptic patterning depends on appropriate patterns of dendritic growth and arborization, we next used electron microscopy (EM) to look for evidence of bipolar and amacrine cell synapses in regions of the *Pten* cKO IPL where ectopic cells and aberrant PKC terminals were observed. Low-magnification EM images confirmed the striking expansion of the IPL and the loose packing of INL cells in adult *Pten* cKO retinas compared to wild-type retinas (Figure 6A,B). In high-magnification EM images, synapses were observed between amacrine and bipolar cells that were ectopically located in the *Pten* cKO IPL (Figure 6C–F). Moreover, the presence of synaptically-located ribbons at aberrant contacts (Figure 6D) suggests that neurotransmission may occur at these sites. Indeed, molecular constituents of bipolar and amacrine cells, albeit somewhat disorganized, were expressed in the IPL of P21 *Pten* cKO retinas. These included VGLUT1 (Figure 6I,I′,J,J′), a pre-synaptic, vesicular glutamate transporter that loads glutamate into a bipolar cell's synaptic vesicles; *PKCα, which labels* glutamatergic *bipolar cell* terminals (Figure 6G,G′,H,H′); and syntaxin, which labels amacrine cell presynaptic terminals (Figure 6G,G′,H,H′). Notably, co-localization of PKCα and syntaxin was detected in ectopic sites in the *Pten* cKO IPL (Figure 6G,G′,H,H′). Misplaced cells can therefore form apparent synaptic contacts in *Pten* cKOs, although from their structure alone, we could not be certain that they were functional.

### *Pten* regulates physiological responses to light

**Figure 7:**
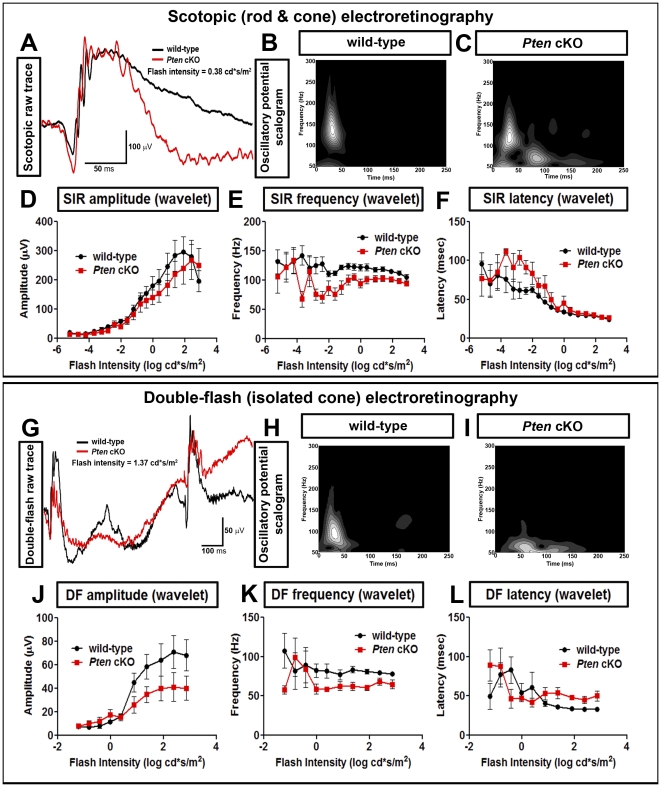
Altered ERG oscillatory potential responses in *Pten* cKO animals. (A–F) Scotopic ERG with representative trace (A; wild-type is black; *Pten* cKO is red) and OP scalogram (B,C) at flash intensity of 0.38 cd*s/m^2^. (D–F) Graphical representation of OP amplitude (D), frequency (E) and latency (F) across 19 steps (−5.22 to 2.86 cd*s/m^2^). (G–L) Double flash ERG with representative trace (G; wild-type is black; *Pten* cKO is red) and OP scalogram (H,I) at flash intensity of 0.38 cd*s/m^2^. (J–L) Graphical representation of OP amplitude (J), frequency (K) and latency (L) across 10 steps (−5.22 to 2.86 cd*s/m^2^).

The presence of an expanded and disorganized IPL and the loss of spatial regularity of certain cellular populations suggested that *Pten* cKO retinae may not be able to respond appropriately to light stimuli. To directly address retinal function, the physiological activity of adult *Pten* cKO retinae was assessed by recording full field ERGs. The photoreceptor (a-wave) and bipolar cell (b-wave) responses (amplitudes and implicit times) to a stepwise series of increasing strengths of flashes of light were similar in both wild-type and *Pten* cKOs under scotopic and photopic adaptation (Figure S3A–F; Table S3). The only significant interaction was a reduction in b-wave amplitude in *Pten* cKOs when activity from cone bipolar cell responses was isolated with a double flash procedure under scotopic adaptation (Figure S3G; Table S3). To investigate the dynamics of amacrine cell-dependent physiological functions [37], we used the Morlet wavelet transformation to simultaneously characterize three oscillatory potential (OP) properties, which can be graphically represented on a scalogram, with amplitude (grey scale, from low to high amplitude in black and white, respectively) plotted relative to ERG frequency (y axis) and latency (x axis) [37]. OPs, which are represented in ERG raw traces and as scalograms (Figure 7A–C,G–I; Figure S4A–C), were strikingly irregular in *Pten* cKO retinae. Quantitatively, while OP amplitudes did not vary between groups, frequencies were significantly reduced under scotopic and photopic adaptation in *Pten* mutants compared to wild-type retinas (Figure 7E,K; Figure S4E; Table S3). A significant interaction was also found in OP amplitudes of *Pten* cKOs when cone-driven responses were isolated with a double flash under scotopic adaptation (Figure 7J; Table S3). Finally, OP latencies were also significantly prolonged under scotopic adaptation in *Pten* mutant mice (Figure 7F; Table S3).

Taken together, these findings reveal abnormal physiological responses in the retinas of *Pten* cKO mice, with light-dependent phase-locking of amacrine cell firing being most severely affected, while other ERG components (i.e., a- and b-waves) were largely spared.

### *Pten* is required for vision-dependent behaviour

**Figure 8:**
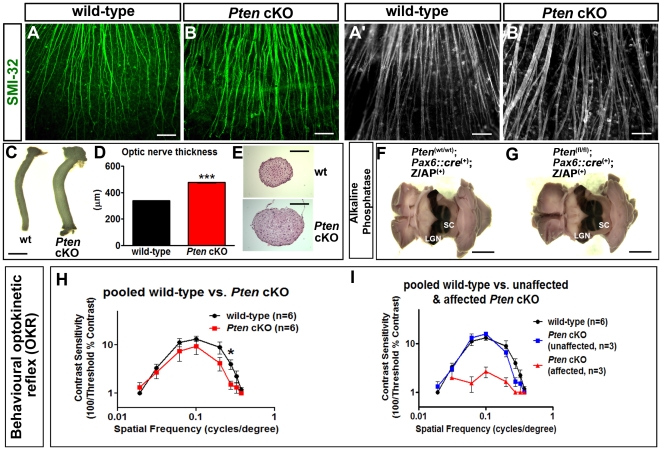
Aberrant RGC fasciculation and subcortical visual responses in *Pten* cKOs. (A–B) Low (A,B) and high (A′,B′) power photomicrographs of SMI-32 labeling of P21 wild-type and *Pten* cKO retinal wholemounts. (C–E) Photomicrographs of wild-type and *Pten* cKO P21 optic nerves (C) and corresponding cross sections stained with hematoxylin-eosin (E). Optic nerve diameters are shown in D. (F,G) AP staining of P21 *Pax6::Cre* ^+^;Z/AP^+^ (“wild-type”; F) and *Pten^f^* ^l/fl^;*Pax6::Cre* ^+^;Z/AP^+^ (*Pten* cKO, G) whole brains with the overlying cortex removed to reveal the visual pathway. The center of the superior colliculus (SC) is unstained as it is innervated by RGCs in the central retina, where cre activity is low. (H,I) Behavioural measures of the optokinetic reflex in adult wild-type and *Pten* cKOs that are either pooled (H) or separated into affected and unaffected groups (I). Scale bars = 300 µm (A,B), 100 µm (A′,B′), 750 µm (C), 200 µm (E), 2.5 mm (F,G).

RGCs are the output neurons of the retina, projecting their axons to visual centers in the brain, including the lateral geniculate nucleus and superior colliculus. Since amacrine cells strongly influence the organization of RGC receptive fields [38], the abnormality of visual processing by amacrine cells in *Pten* mutants could affect visual behaviour. To assess whether *Pten* is required for higher-order visual functioning, we first determined whether RGC axons projected to their appropriate central targets in the absence of *Pten* function. RGC axons were first labelled with SMI-32 in P21 retinal flatmounts, and the fascicles were observed to be thicker and more bundled in *Pten* cKO *vs* wild-type retinas (Figure 8A,A′,B,B′). *Pten* cKO optic nerve diameters were also increased compared to controls (Figure 8C–E). These results suggest that, like the RGC cell bodies themselves (Figure 2Q–S), their optic axons are hypertrophic, although we cannot rule out the possibility that an increase in axon number also contributes, particularly since retinal area (Figure 4C) increases almost as much as does the increase in the cross-sectional area of the optic nerve (Figure 8C–E). Regardless of the cause of the optic nerve hypertrophy, by using a Z/AP dual reporter to label *Pax6::cre*-recombined (i.e., AP^+^) RGC axons [29], [30], [39], we observed that the AP-labeled optic tract innervated the dorsal lateral geniculate nucleus (dLGN) and superior colliculus (SC) similarly in P21 wild-type and *Pten* cKOs (Figure 8F,G). Thus, deletion of *Pten* in RGCs does not influence the specificity of their targeting to these two main retinofugal targets. Indeed, the AP-negative strip associated with central RGCs that did not undergo cre-mediated recombination (Figure 1H) was apparent across the medio-lateral axis of the superior colliculus in the *Pten* cKO retina, as it is in the wild-type (Figure 8F,G), indicating no gross disruption of retinotopic order across the surface of the superior colliculus.

To determine whether the spatiotemporal processing of visual information and its transmission to the brain were altered in *Pten* cKO animals, we analyzed the optokinetic reflex (OKR), a subcortical motor response to moving stripe patterns that is a reliable and quantitative behavioural indicator of some aspects of retinal function [40]. OKRs were measured using a virtual cylinder that displayed vertical black and white stripes of varying dimensions and contrasts, rotated at different speeds. In wild-type adult mice, maximum contrast sensitivities were uniformly ∼15.8 (threshold contrast = 6.3%) at the optimal spatial frequencies (0.061 and 0.1 cycles/degree (c/d)), while the lower and upper limits of spatial frequencies that evoked an OKR at 100% contrast were 0.019 and 0.375 c/d, respectively (Figure 8H). Average contrast sensitivities of wild-type and *Pten* cKO animals at 0.275 c/d were significantly different (n = 6 each; P = 0.034; Table S4). However, the distribution of contrast sensitivities in *Pten* cKOs was bimodal, falling into a severely affected and a relatively unaffected group (n = 3 each). The OKR contrast sensitivities of “unaffected” *Pten* cKO mice were indistinguishable from those of wild-type mice, except for a mild attenuation of contrast sensitivity and lowering of acuity at the highest spatial frequencies (Figure 8I). The OKR contrast sensitivities of “affected” *Pten* cKOs were significantly different from those of either wild-type (P<0.001) or “unaffected” *Pten* cKOs (P = 0.003; Table S4), reaching a maximum of only 2 (threshold contrast = 50%) at the optimum spatial frequency, 0.1 c/d, and could be elicited reliably only from 0.061–0.200 c/d (Figure 8I).

Thus, some *Pten* cKO mutants have a diminished capacity to respond to motion of a global stripe pattern, suggesting that the inner-retinal (bipolar-amacrine-ganglion cell) circuits that mediate contrast sensitivity in this behavioural paradigm are impaired.

### *Dscam* does not regulate Pten/PI3K signalling in the retina

**Figure 9:**
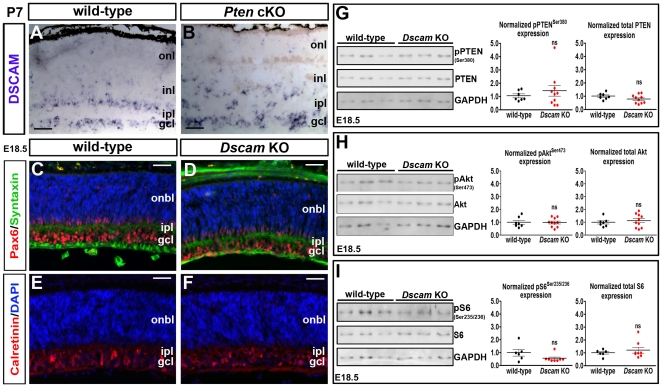
Interactions between *Pten* and *Dscam*. (A,B) Distribution of *Dscam* transcripts in P7 wild-type (A) and *Pten* cKO (B) retinae. (C–F) Labeling of E18.5 wild-type and *Dscam* KO retinae with Pax6 (red)/syntaxin (green; C,D) and calretinin (red; E,F). Blue is DAPI counterstain. (G–I) Western blotting and densitometry for PTEN and pPTEN^Ser380^ (G), total Akt and pAkt^Ser473^ (H), and total S6 and pS6^Ser235/236^ (I) in E18.5 wild-type and *Dscam* mutants. gcl, ganglion cell layer; inl, inner nuclear layer; ipl, inner plexiform layer; onl, outer nuclear layer; opl, outer plexiform layer. Scale bars = 50 µm.

Since PTEN is an intracellular signaling molecule, it seemed likely itself to be regulated by extrinsic signals. For that reason, we were struck by the similarities between *Pten* and *Dscam* mutant retinas, both of which develop a markedly thickened IPL and display aberrant fasciculation and mosaic patterning of subsets of amacrine cells [9], [11]. To test for regulatory interactions between *Pten* and *Dscam*, we first determined whether retinal expression of *Dscam* was altered in the absence of *Pten* expression. *Dscam* is expressed in amacrine cells and RGCs in the INL and GCL [9], [11], as shown here in P7 wild-type retinas (Figure 9A). In P7 *Pten* cKO retinas, *Dscam* transcripts were similarly detected in the INL and GCL, but expression was also detected in ectopic cells in the IPL (Figure 9B). Thus, the maintenance of *Dscam* expression in *Pten* cKO retinas was not itself sufficient to prevent amacrine cells and/or RGCs from aberrantly migrating into the IPL. Moreover, DSCAM does not prevent amacrine cell processes from fasciculating in the IPL in *Pten* cKO retinas, even though DSCAM is thought to prevent such homotypic adhesion [10].

Next we asked the converse question, examining whether PTEN protein and/or activity levels were altered in *Dscam* mutant retinas. For this purpose we acquired *Dscam* null mutants, most of which die immediately after birth [41], contrasting to spontaneous *Dscam* mutants, which survive postnatally [9], [11], [12]. Amacrine cell spacing and projection defects were not yet apparent in *Dscam* mutants at E18.5, as revealed by Pax6, syntaxin and calretinin immunolabeling (Figure 9C–F). Notably, amacrine cell defects were also not yet apparent in *Pten* cKOs at E18.5 (data not shown). Nevertheless, we reasoned that E18.5 was an appropriate stage to study for our purposes, as amacrine cells are actively migrating [42] and have begun to innervate the IPL [43] at this stage in wild-type mice. Western blot analysis of E18.5 retinal tissue revealed that PTEN, pPTEN^Ser380^, pAkt^Ser473^ and pS6 levels were not significantly different in wild-type and *Dscam* mutant retinas (Figure 9G–I).

The lack of alterations in PI3K signalling components, including PTEN, in *Dscam* mutant retinas, suggests that that PI3K signaling is not regulated by DSCAM in the retina, at least at E18.5, when amacrine cells are migrating and the IPL is beginning to form.

## Discussion

The acquisition of a functional retinal architecture requires the coordination of multiple events, including the control of cell growth, migration, neurite arborization and synaptogenesis. How these events are coordinated in such a way as to ensure proper neuronal positioning, both within individual retinal cell populations and across the retinal layers, is poorly understood. Here we have identified the PTEN phosphatase as a critical regulator of retinal tissue morphogenesis, identifying roles for this phosphatase in mosaically-patterned RGCs, amacrine and horizontal cells. We found that while *Pten* restricts the growth of each of these cell types, it has cell type-specific roles in regulating the radial and tangential migration and neurite arborization patterns of each cell population. In the radial axis, we detected ectopic RGCs and amacrine cells in the IPL, while in the tangential plane, TH^+^ amacrine cells are particularly sensitive to the loss of *Pten*, displaying defects in the regularity of their patterned somal distributions, as well as aberrant fasciculation of their processes. The dendrites of *Pten* mutant melanopsin^+^ ipRGCs likewise show aberrant fasciculation, but colonize their appropriate strata within the IPL sublaminae. Similarly, the spacing of calbindin^+^ horizontal cells is disrupted in the tangential dimension in *Pten* mutant retinas but the positioning of their somata across the radial axis of the retina is not perturbed; nor is the stratification of their processes affected. Finally, we show that PTEN/PI3K signaling is not altered in *Dscam* mutant retinas, suggesting that PTEN does not function downstream of this cellular adhesion pathway to regulate amacrine and RGC cell and neurite spacing, at least during the embryonic period. Taken together, these data indicate that distinct molecular controls govern the radial and tangential migration of retinal cell bodies to their final destinations, where they establish specific arborization patterns and synaptic connections, and implicate *Pten* as a critical component of several of these pathways.

### *Pten* regulates cell and neurite growth in the retina

*Pten* is a well-known negative regulator of cellular growth, as evidenced by its designation as a tumor suppressor gene. Accordingly, in humans, germline *Pten* mutations are also associated with hamartoma tumour syndromes (e.g., Lhermitte–Duclos disease, Cowden syndrome), which are characterized by tumour-like clusters of overgrown, differentiated cells [44]. Moreover, tissue-specific mutations of *Pten* in the mouse result in hyperplasia in multiple organs, including the pancreas, hippocampus, neocortex and cerebellum [18], [22], [45], [46]. However, *Pten's* role in growth control is tissue-specific, as it is not required to prevent overgrowth of thymocytes and fibroblasts [47]. Here we report that *Pten* cKO causes RGC, horizontal and amacrine cell hypertrophy, and suggest that this is due to elevated mTOR signalling [31]. We also observe a progressive thickening of the IPL and optic nerve from P7 to adulthood in *Pten* cKOs, suggesting a continuity of growth of amacrine/RGC processes, similar to CNS hyperplastic defects reported in other *Pten* cKOs [24], [26]. Notably, such overgrowth can be beneficial as optic nerve regeneration is enhanced when *Pten* is knocked down in damaged optic nerves [48], resulting in some re-innervation of the dorsal LGN [49].

In the context of normal development, cellular hypertrophy is obviously not beneficial, and likely contributes to the aberrant tissue morphogenesis observed in *Pten* cKO retinas. For instance, the continual growth of amacrine cells, including their processes, likely contributes to the aberrant patterning of the IPL we observe in *Pten* cKO retinas. Nevertheless, we do not believe that cellular hypertrophy is primarily responsible for all defects observed in *Pten* cKO retinas, for the following reasons: 1) When retinal cell sizes increase, one would expect a uniform increase in Voronoi domain size and nearest neighbor distances. Instead, what we observe in *Pten* cKO retinas is a decrease in the regularity of these parameters, indicating that the normal spatial relationships between TH^+^ amacrine cells and calbindin^+^ horizontal cells are disordered. 2) While ipRGCs also increase in size in *Pten* cKO retinas, their dendrites target the correct sublamina in the IPL, and aberrant dendritic fasciculation is only observed in the tangential plane. Moreover, RGC axons still target their appropriate retino-recipient nuclei, the LGN and superior colliculus, and apparently do so in a normal retinotopic manner, at least at the gross level.

The cell type-specific effects of *Pten* in the context of retinal cell migration and neurite patterning are discussed in more detail in the following sections.

### *Pten* regulates retinal cell migration in a cell type-specific manner

Cones, RGCs, horizontal and amacrine cells migrate radially to form layers, and then disperse in the tangential plane to form non-random cellular mosaics [33]. Conversely, rod and bipolar cell migration is only regulated in the radial axis [33]. We found that some RGCs and amacrine cells were ectopically positioned in the *Pten* cKO IPL, consistent with *Pten'*s known role in regulating radial migration in other regions of the central nervous system [17]–[19]. In contrast, we did not find any evidence for aberrant positioning of horizontal cells across the depth of *Pten* cKO retinas. However, TH^+^ amacrine cells and calbindin^+^ horizontal cells were aberrantly dispersed in the tangential axis in *Pten* cKOs. Fundamental differences in how RGC, horizontal and amacrine cells migrate may explain differences in *Pten*-dependency. Upon differentiation, amacrine cells lose their apico-basal contacts and are thus more sensitive to environmental cues in their migratory path compared to RGCs, which retain a basal attachment (presumptive axon) that acts as a “tether” to pull RGCs into the GCL [1]. Similarly, horizontal cell progenitors lose their apical and basal contacts and divide in non-apical positions before going to their final, definitive position, making them more sensitive to environmental cues [50]. Thus, amacrine cells and horizontal cells may be most sensitive to the loss of *Pten* if the loss of this signalling molecule results in an impaired ability to “sense” the proximity of like-neighbours, or an inherent inability to follow tangential migratory cues [51]. This raises the question of what the extrinsic cue(s) might be that require PTEN to transduce their signal?

Aberrant retinal spacing in *Pten* cKOs may arise because of defective intercellular adhesion, a process that *Pten* regulates in the retinal pigment epithelium (RPE) [52]. Indeed, the cell spacing defects in *Pten* cKO retinas most closely resemble those observed following the mutation of several adhesion molecules. For example, mutations in zebrafish *N-cadherin* also result in hypertrophic RGC and amacrine cell bodies and processes that are aberrantly patterned [53], [54]. Here we investigated the relationship between *Pten* and DSCAM, given the cellular specificity of *Pten* cKO phenotypes most closely phenocopy those observed in *Dscam* mutants [9]–[12]. While DSCAM's role in cell adhesion is evolutionarily conserved, how it functions has changed. *Drosophila Dscam1* undergoes extensive alternative splicing, resulting in cell type-specific expression of multiple, unique isoforms that prevent homophilic (self-self) associations [55], [56]. In contrast, mammalian *Dscam* is not extensively alternatively spliced, such that neighboring retinal cells whose dendritic arborizations overlap can express the same isoform [10], [57]. DSCAM therefore cannot act as a direct repulsive cue during mosaic patterning. Instead, it has been suggested that the aberrant clustering of amacrine cells and RGCs in *Dscam* mutant retinas arises because DSCAM is required to block unknown adhesive pathways that are normally silenced. Unmasking of these adhesive pathways in *Dscam* KOs results in aberrant mosaicism and neurite fasciculation [10], [57].

Interestingly, the aberrantly positioned retinal cells in the *Pten* cKO IPL continue to express *Dscam*, suggesting that the normal “anti-adhesive” properties of DSCAM are not sufficient to prevent cellular mispositioning, at least in the absence of *Pten*. Defects in cell spacing and neuritic differentiation as well as an expanded IPL similar to those in the *Dscam* mutant retina have also been detected in the *Bax* KO retina, where there is similarly no modulation of *Dscam* expression [13]. However, it is important to note that there are also fundamental differences in the aberrant mosaic patterns observed in *Pten* cKO (this study), *Dscam* KO [9]–[13] and *Bax* KO [13] retinas. Most notably, while we have observed a skewed distribution towards larger distances between nearest neighbors and Voronoi domain sizes for both calbindin^+^ horizontal cells and TH^+^ amacrine cells in *Pten* cKO retinas, these parameters are instead skewed towards the smaller end in *Dscam* KO and *Bax* KO retinas. That is to say, there is an increased clustering of amacrine cells in *Dscam* KO and *Bax* KO retinas, while conversely; we observe an overall increase in the spacing of these cells in *Pten* cKO retinas (albeit one that similarly degrades mosaic regularity). Possible causes for the increased distances between homotypic cells in *Pten* cKO retinas may include the reduction in horizontal and amacrine cell numbers, or because these cell types increase in size. However, it remains formally possible that these defects may also reflect aberrant migratory properties of *Pten* mutant retinal cells in the tangential plane. Indeed, tangential dispersion is a key determinant in the establishment of horizontal, cholinergic and ganglion cell mosaics [7], although it appears to play a minimal role in establishing the mosaics of TH^+^ amacrine cells [34].

Future studies will be required to establish the precise role that *Pten* plays in establishing retinal cell mosaics. Nevertheless, we can conclude that DSCAM and PTEN may function in independent pathways as *Dscam* expression is maintained in *Pten* cKO retinas, and PTEN/PI3K protein and activity levels are unperturbed in *Dscam* mutant retinas, at least at E18.5. However, PTEN also has PI3K-independent nuclear functions [58], suggesting that *Pten* may function in unknown ways to contribute to *Dscam*-mediated retinal cell spacing.

### *Pten* regulates laminar patterning of the IPL

PI3K and PTEN activity are high in the P7 IPL, during the period when RGC, amacrine cell, and bipolar cell neurites are actively innervating IPL sublamina in a highly-patterned array. Accordingly, we found that *Pten* is required for IPL stratification. Specifically, the targeting of most amacrine cell processes and bipolar cell axons to appropriate IPL sublamina is severely disrupted in *Pten* cKOs, consistent with recent findings obtained by a different group using a *Chx10::cre* driver line [28]. Two exceptions to this rule are the ipRGC dendrites and TH^+^ amacrine cell processes, both of which normally targeted substratum 1 in *Pten* cKO retinae. This finding was particularly surprising given the hyper-fasciculation of ipRGC dendrites and TH^+^ amacrine cell processes in the tangential plane.

Dscam and the related IgSF molecules DscamL and Sidekick1/2 also participate in IPL sublamination [12], [14], further supporting the similarity of PTEN and adhesion molecule function in retinal patterning. In addition, *PlexinA4/Sema6a, Sema5a/Sema5b*, and *PlexinA1/PlexinA3* double mutants each display similar, but also each unique types of IPL patterning deficits to those seen in *Pten* cKOs, with the exception that they do not have an expanded IPL [16]. While intracellular signalling cascades that operate downstream of Sema5a, Sema5b, Sema6a or PlexinA1, PlexinA3, PlexinA4 have yet to be characterized in the retina, PlexinB1/Sema4D have been shown to regulate PTEN and PI3K activities in a context-dependent manner [59], [60]. It is tempting to speculate that PTEN activity may be regulated by some combination of Plexin/Sema receptor signalling in the retina, in such a way as to control the targeting of amacrine cell processes in the IPL without affecting their growth.

### *Pten*'s role in regulating visual physiology and behaviour

By conducting a thorough ERG analysis in *Pten* cKO mice, we found that photoreceptor (A-wave) and bipolar cell (B-wave) function is relatively unaffected in these mutant mice. However, further analyses using a continuous Morlet analysis of ERG responses over a wide range of stimulus intensities allowed us to define the dynamics of OP firing in *Pten* cKO mice. These studies revealed a dysregulation of light-driven phase locking in *Pten* cKO mice, which we infer to mean that *Pten* is required for the synchronous firing of amacrine cells. In contrast, a recent study in a different conditional *Pten* cKO model reported no changes in OPs with the exception of increased amplitudes only under photopic adaptation, possibly because they only performed limited amplitude measurements of filtered ERG traces (50–170 Hz) at a single flash strength [28]. Other studies have similarly suggested that *Pten* regulates neural circuitry function. For instance, *in vivo* knockdown of *Pten* in adult mouse hippocampus results in an increased excitatory drive onto dentate granule cells [61]. Another study has shown that *Pten* also regulates proper long-term potentiation and depression in the hippocampus, independently of its effects on cell morphology and migration [62]. Interestingly, *Dscam* null mutants similarly display a loss of synchronous firing of pre-inspiratory neurons in the medulla, the site of the rhythm generator that controls respiration, accounting for the premature death of these animals [41]. However, while the ERG of dark-adapted mice was previously tested in spontaneous *Dscam* mutants [11], it remains to be determined whether OPs or OKRs are aberrant in them.

In addition to the aberrant retinal physiology in *Pten* cKO mice, we further suggest that these defects in amacrine cell function also influence visual behaviour. Indeed, evoking an OKR is dependent upon image-processing by directionally selective RGCs [40], the activity of which is influenced by amacrine cells [63], and we found that approximately half of *Pten* cKO mice display OKR defects. These OKR defects are unlikely due to a perturbation of the visual pathway outside of the retina. Indeed, despite the striking increase in *Pten* cKO optic nerve diameter, gross overall connectivity (i.e., RGC targeting of the SC and LGN) of the visual pathway was apparently unaffected by the loss of this signaling molecule. Future experiments will be needed to determine what underlies the increased optic nerve thickness in *Pten* cKO mice, whether it arises due to an increase in numbers of RGC axons, hypertrophy of individual RGC axons, increased fasciculation, or enhanced myelination.

It is currently unknown why some *Pten* cKO mice have an apparently normal capacity to respond to motion of a global stripe pattern, a behaviour that depends on inner-retinal circuitry (bipolar-amacrine-ganglion cell), which was strikingly disrupted in these mutants. However, we suggest that the disruption in IPL patterning in *Pten* cKO retinas is likely due to the progressive overgrowth of amacrine cell processes and RGC dendrites, and it may be that prior to their overgrowth, these neurons establish some appropriate connections. Future experiments will be needed to determine if some *Pten* cKO mice still contain some level of appropriate connectivity and/or physiological activity between specific populations of amacrine cells and RGCs that would allow for proper OKR responses.

In summary, we have identified cell type-specific functions for *Pten* in regulating migration and neurite arborization in a subset of retinal RGCs, horizontal cells and amacrine cells. This is an unexpected level of cellular specificity, given that PTEN and other signalling molecules are thought to be ubiquitously expressed and to have pleiotropic functions. Instead, our data support specific roles for *Pten* in individual retinal cell populations, specifically those that form retinal mosaics. However, within each of these cell populations, *Pten* may have multiple roles, regulating not only cell growth, but also migration, neurite arborisation and neuronal function. The future goal will be to determine how PTEN function is regulated and functions within each of these cell populations to carry out each of its precise roles.

## Materials and Methods

### Animals

All animal procedures were compliant with the Guidelines of the Canadian Council of Animal Care (CCAC) and were approved by the University of Calgary Animal Care Committee under animal protocol M08006. *Pax6::cre* [29] and Z/AP [30] transgenes were maintained on a CD1 background and genotyped as described. The *Pten* ^fl^ allele was maintained on a mixed C57/Bl6/SV129 background and genotyped as described [18]. The *Dscam* mutant allele was genotyped as described [41].

### RNA in situ hybridization, immunofluorescence, histology and electron microscopy

RNA *in situ* hybridization was performed as previously described [64] using a *Dscam* probe. Section immunofluorescence was conducted as previously described [42]. For staining of retinal flatmounts, eyes were dissected and the cornea, RPE, lens and blood vessels were removed before flattening the retina (GCL up) on Nucleopore® track-etched membranes (Whatman #110409). Flattened retinae were fixed with 1 ml of 4% paraformaldehyde (PFA)/1× phosphate buffered saline (PBS) for 1 hour at 4°C, and washed briefly in 1× PBS before blocking and incubation in 1° antibody in blocking solution for 5–7 days at 4°C on a rocker. Subsequent steps and antibodies were as described for section immunostaining [42]. Primary antibodies for immunostaining were to: PTEN (1∶100, Cell Signalling #9559), TH (1∶50, Santa Cruz #sc-14007), melanopsin (1∶2500 wholemount, 1∶5000 frozen sections, Advanced Targeting Systems #AB-N38), SMI-32 (1∶250, Covance #SMI-32R), calbindin-D (1∶500, Sigma #C-9848), ChAT (1∶250, Chemicon #AB144P). For birthdating studies, intraperitoneal injections of 100 µg/g body weight BrdU (Sigma) were performed on timed staged pregnant females. For BrdU immunolabeling, sections were treated with 2 N HCl for 15 min at 37°C prior to processing. For histological staining, whole eyes were placed in Bouin's fixative and processed for paraffin sectioning and hematoxylin-eosin staining as described [65]. AP and β-gal staining was performed as described [30]. For EM, eyes were dissected and processed as described [66].

### Western blotting

Retinae were lysed and Western blots performed as previously described [42]. Primary antibodies were to: pAktSer473 (1∶1000, Cell Signalling #4060), total Akt (1∶2000, Cell Signalling #9272), PTEN (1∶1000, Cell Signalling #9559), pS6^Ser235/236^ (1∶1000, Cell Signalling #4856), S6 (1∶1000, Cell Signalling #2217), β-actin (1∶5000, Abcam #8227), GAPDH (1∶5000, Cell Signalling #2118).

### ERG analyses

For ERG analyses, briefly, animals were dark-adapted for one hour and prepared for recordings under dim red light. Stimulation and acquisition were achieved by a commercial system (Espion E^2^ from Diagnosys LLC; flash duration 10 µs, bandpass filtering 0.3 Hz–3 Khz). ERGs were conducted as previously described [67], [68]. Scotopic intensity responses consisted of single flash presentations at 19 increasing flash strengths from −5.22 to 2.86 log cds/m^2^. For double flash ERGs, a probe flash (covering −1.6 to 2.9 log cds/m^2^) was presented 0.8 s after a conditioning flash (1.4 log cds/m^2^). Finally, photopic intensity responses (30 cd/m^2^ background light) consisted of 11 increasing flash strengths ranging from −1.6 to 2.9 log cds/m^2^. In addition to analysis of the a- and b-waves (amplitude and implicit time), three properties of the OPs (amplitude, frequency and latency) were quantified using Morlet wavelet transform [69].

### Optokinetic Testing

Optokinetic testing was conducted as described [40]. Briefly, mice were placed on a platform centered in a chamber surrounded by four −17″ monitors, and the reflexive optokinetic response (OKR; head-turning) was elicited by a virtual-cylinder sine-wave grating moving leftward or rightward at constant velocity (OptoMotry™, Cerebral Mechanics Inc., Lethbridge, AB, Canada). The drift speed was constant at 12 degrees/second (d/s), contrast (Michelson contrast) ranged from 0 to 100%, and spatial frequencies ranged from 0.019 to 0.4 cycles/degree (c/d). Contrast threshold was defined as the lowest contrast at which an OKR could be elicited reliably by a grating of a given spatial frequency; contrast sensitivity was defined as the reciprocal of contrast threshold, in arbitrary units (100/[threshold % contrast]); and acuity was defined as the highest spatial frequency that reliably elicited an optokinetic response at 100% contrast (spatial frequency at contrast sensitivity = 1).

### Measurements and statistical analysis

Photomicrographs of cells expressing cell type-specific marker(s) were used to count cell number/field. In all experiments, cells were counted from a minimum of 3 retinas. Somal areas of immunolabeled cells were calculated using Photoshop CS3 (Adobe Systems, San Jose, CA). The Delunay-Voronoi plugin for Image J (http://rsbweb.nih.gov/ij/) was used for collecting the X-Y coordinates of TH^+^ amacrine and calbindin^+^ horizontal cells. These were exported to a customized program that computed the Voronoi tessellation of the field and the near neighbour distances of each individual cell (excluding cells with Voronoi domains that intersected the boundaries of a field), from which the regularity indices of the distribution of Voronoi areas and nearest neighbour distances were calculated (mean/standard deviation) [8]. Statistical significance for cell counts, cellular area, cellular spacing, western blotting densitometry, and optic nerve thickness were calculated using two-way Student's t-tests using GraphPad Prism Software version 5.0 (GraphPad Software Inc., La Jolla, CA). All analyses were performed on a minimum of three eyes/genotype, and 3–10 photomicrographs/eye. See Tables S1, S2, S3, S4 for details and exact p-values for all tests. Analyses for ERGs was performed using repeated measures ANOVA (rmANOVA) in SPSS 17.0 (SPSS Inc. Chicago, IL, USA) with mouse genotype (wild-type versus *Pten* cKO) being used as the between-subject factor for either a main statistical difference or an interaction effect. See Table S3 for details and exact p-values for all tests. OKR behavioural analyses were carried out using a Mann-Whitney U-test for pooled wild-type versus *Pten* cKO mice and a linear mixed model (LMM) was used to analyse data when *Pten* cKO mice were placed in affected or unaffected categories. Genotype is included in the model as a fixed factor with 3 levels (wild-type versus affected or unaffected *Pten* cKO) in OKR data analysis. Post-hoc pair-wise comparison between genotypes was carried out using Least Significant Difference (LSD) with p-values adjusted for multiple comparisons. See Table S4 for details and exact p-values for all tests. All graphs were generated using GraphPad Prism Software version 5.0 with error bars representing the standard error of the mean (s.e.m.).

## Supporting Information

Figure S1 **Ectopic RGC and amacrine cells in** ***Pten*** **cKO retinae.** (A–D) Brn3a (A,B) and Pax6 (C,D) immunostaining of P7 retinal cross-sections in wild-type (A,C) and *Pten* cKO (B,D) retinas. Blue is DAPI counterstain. gcl, ganglion cell layer; inl, inner nuclear layer; ipl, inner plexiform layer; onl, outer nuclear layer; opl, outer plexiform layer. Scale bars = 50 µm.
(TIF)

Figure S2 **Apoptosis is unaltered in** ***Pten*** **cKO retinae.** (A–L) Active caspase-3 immunostaining at E15.5 (A–D′), P0 (E–H′) and P7 (I–L′) in wild-type (A–B′,E–F′,I–J′) and *Pten* cKO (C–D′,G–H′,K–L′) retinas. Blue is DAPI counterstain. gcl, ganglion cell layer; inl, inner nuclear layer; le, lens; onbl, outer neuroblast layer; onl, outer nuclear layer; re, retina. Scale bars = 300 µm (A,C), 600 µm (E,G,I,K).
(TIF)

Figure S3 **Scotopic, photopic and double-flash ERG analysis of A-waves and B-waves in adult** ***Pten*** **cKO mice.** (A–H) Scotopic A-wave amplitudes (A) and implicit times (B) in wild-type and *Pten* cKO mice. Scotopic B-wave amplitudes (C) and implicit times (D) in wild-type and *Pten* cKO mice. Photopic B-wave amplitudes (E) and implicit times (F) in wild-type and *Pten* cKO mice. Double-flash B-wave amplitudes (G) and implicit times (H) in wild-type and *Pten* cKO mice (wild-type is black; *Pten* cKO is red).
(TIF)

Figure S4 **Photopic ERG oscillatory potential responses in** ***Pten*** **cKO animals.** (A–F) Photopic ERG representative trace (A; wild-type is black; *Pten* cKO is red) and representative OP scalograms (B,C) at the flash intensity of 2.86 cd*s/m^2^. (D–F) Graphical representation of OP amplitude (D), frequency (E) and latency (F) across 11 steps (−1.63 to 2.86 cd*s/m^2^).
(TIF)

Table S1 Comparison of retinal cell sizes in wild-type and *Pten* cKO retinae.
(DOC)

Table S2 Analysis of horizontal and amacrine cell spacing and wholemount cell counts in P21 wild-type and *Pten* cKO retinal flatmounts.
(DOC)

Table S3 Analysis of electroretinogram responses in adult wild-type and *Pten* cKO mice.
(DOC)

Table S4 Analysis of optokinetic contrast sensitivity responses in adult wild-type and *Pten* cKO mice.
(DOC)
