# Supplementary figures and images for: Cell-Type Specific Roles for PTEN in Establishing a Functional Retinal Architecture
Source: PLoS One. 2012 Mar 5;7(3):e32795. doi: 10.1371/journal.pone.0032795 (PMC3293905; doi:10.1371/journal.pone.0032795)

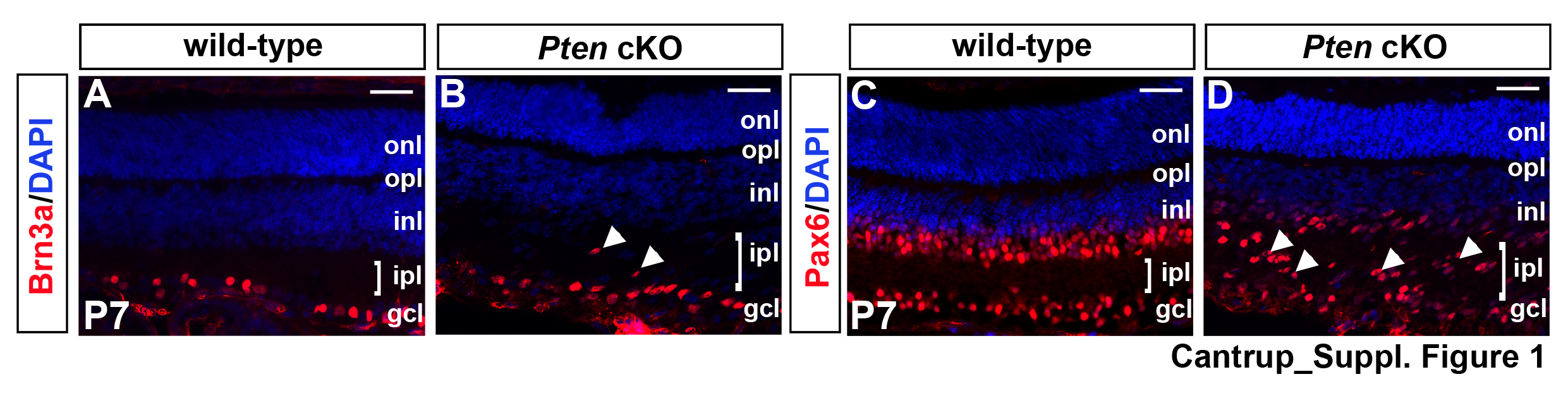

Supplement: Figure S1 — Ectopic RGC and amacrine cells in Pten cKO retinae. (A–D) Brn3a (A,B) and Pax6 (C,D) immunostaining of P7 retinal cross-sections in wild-type (A,C) and Pten cKO (B,D) retinas. Blue is DAPI counterstain. gcl, ganglion cell layer; inl, inner nuclear layer; ipl, inner plexiform layer; onl, outer nuclear layer; opl, outer plexiform layer. Scale bars = 50 µm. (TIF) [file pone.0032795.s001.tif]

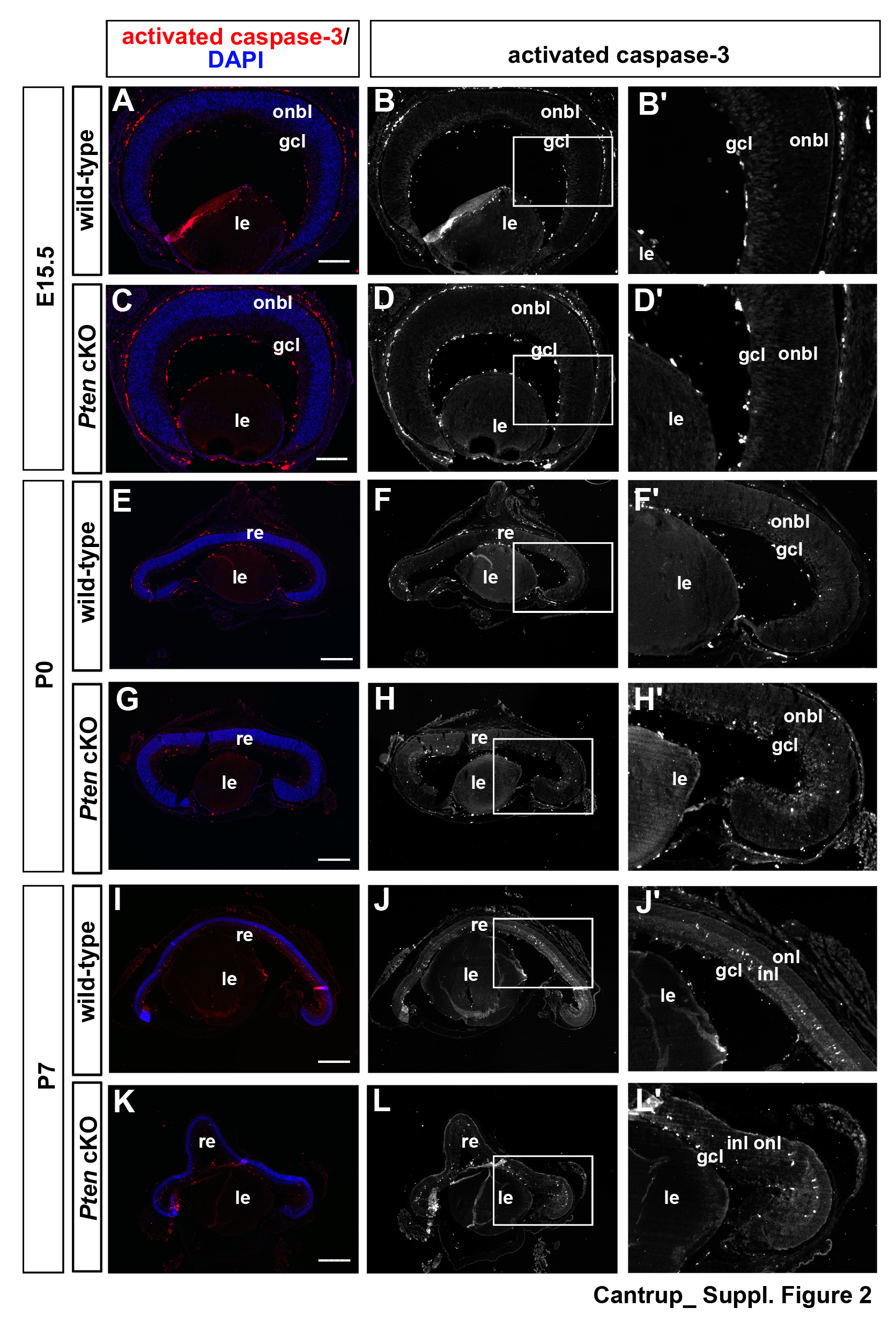

Supplement: Figure S2 — Apoptosis is unaltered in Pten cKO retinae. (A–L) Active caspase-3 immunostaining at E15.5 (A–D′), P0 (E–H′) and P7 (I–L′) in wild-type (A–B′,E–F′,I–J′) and Pten cKO (C–D′,G–H′,K–L′) retinas. Blue is DAPI counterstain. gcl, ganglion cell layer; inl, inner nuclear layer; le, lens; onbl, outer neuroblast layer; onl, outer nuclear layer; re, retina. Scale bars = 300 µm (A,C), 600 µm (E,G,I,K). (TIF) [file pone.0032795.s002.tif]

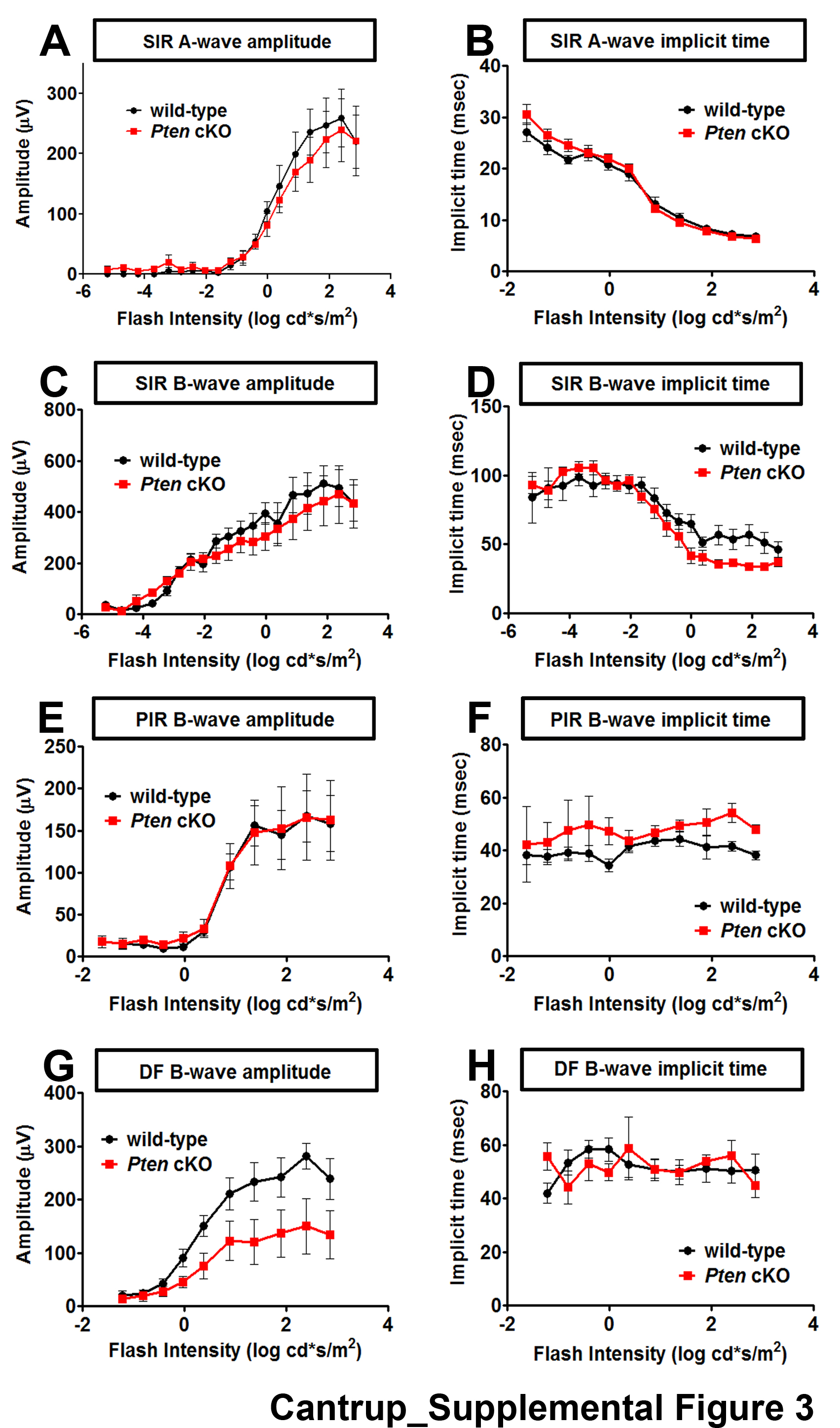

Supplement: Figure S3 — Scotopic, photopic and double-flash ERG analysis of A-waves and B-waves in adult Pten cKO mice. (A–H) Scotopic A-wave amplitudes (A) and implicit times (B) in wild-type and Pten cKO mice. Scotopic B-wave amplitudes (C) and implicit times (D) in wild-type and Pten cKO mice. Photopic B-wave amplitudes (E) and implicit times (F) in wild-type and Pten cKO mice. Double-flash B-wave amplitudes (G) and implicit times (H) in wild-type and Pten cKO mice (wild-type is black; Pten cKO is red). (TIF) [file pone.0032795.s003.tif]

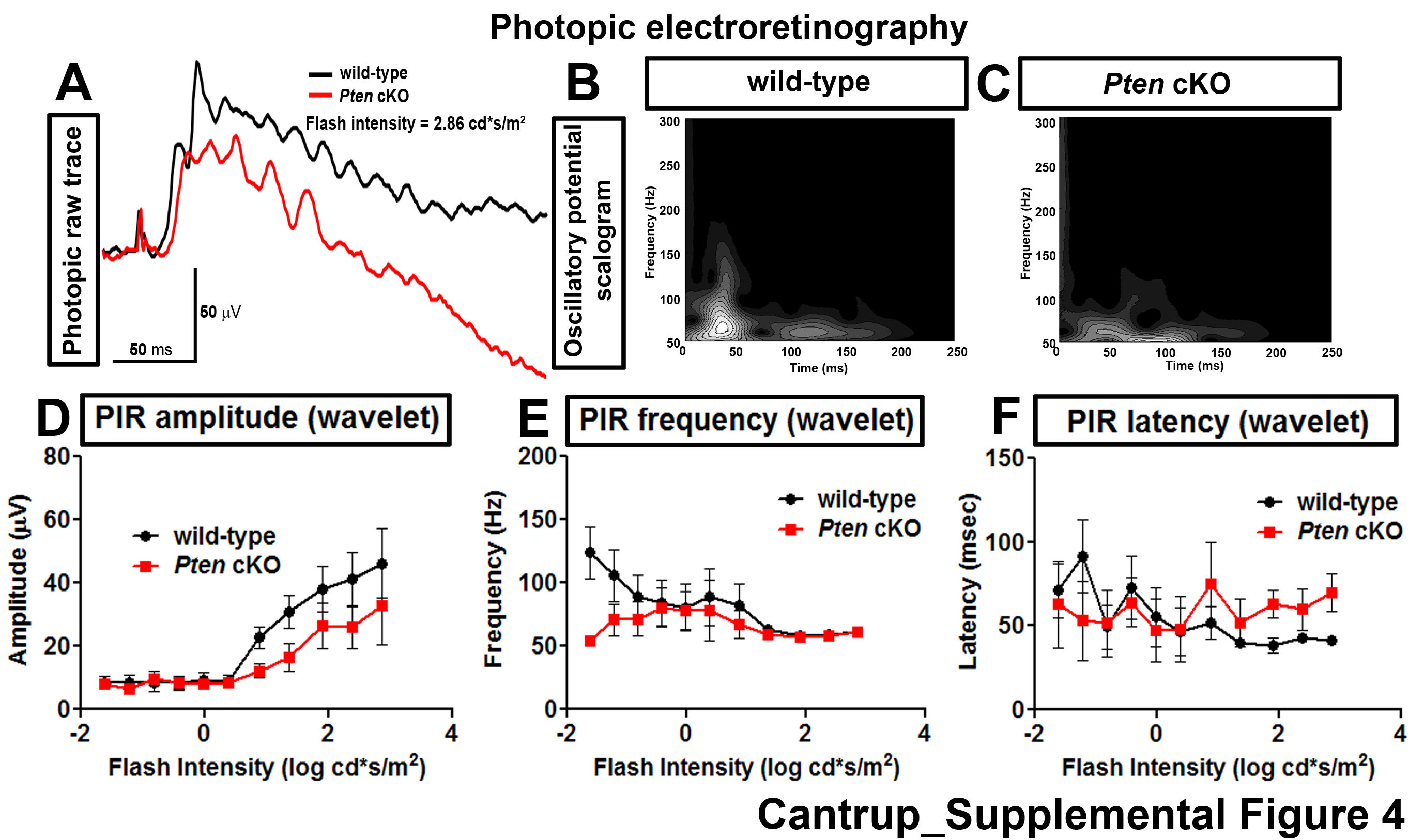

Supplement: Figure S4 — Photopic ERG oscillatory potential responses in Pten cKO animals. (A–F) Photopic ERG representative trace (A; wild-type is black; Pten cKO is red) and representative OP scalograms (B,C) at the flash intensity of 2.86 cd*s/m2. (D–F) Graphical representation of OP amplitude (D), frequency (E) and latency (F) across 11 steps (−1.63 to 2.86 cd*s/m2). (TIF) [file pone.0032795.s004.tif]
