# Supplementary material for: Cell-Type Specific Roles for PTEN in Establishing a Functional Retinal Architecture
Source: PLoS One. 2012 Mar 5;7(3):e32795. doi: 10.1371/journal.pone.0032795 (PMC3293905; doi:10.1371/journal.pone.0032795)
Supplement: Table S1 — Comparison of retinal cell sizes in wild-type and Pten cKO retinae. (DOC) [file pone.0032795.s005.doc]

**Table S1:** **Comparison of retinal cell sizes in wild-type and *Pten* cKO retinae.**

|  | wildtype | *Pten* cKO | Fold-Difference  (wild-type/*Pten* cKO) | P value |
| --- | --- | --- | --- | --- |
| Calbindin^+^ horizontal cells in P7 sections | 3488.5±137.2 pixels  (surface area),  N=6 eyes, 118 cells | 5233.0±369.0 pixels  (rel. µm^2^),  N=6 eyes  143 cells | 1.5-fold increase | p=0.001 |
| Calbindin^+^ horizontal cells in P21 flatmounts | 2812.4±162.8 pixels  (rel. µm^2^),  N=7 eyes, 856 cells | 5854.9±811.2 pixels  (rel. µm^2^),  N=6 eyes  497 cells | 2.1-fold increase | p=0.002 |
| ChAT^+^ amacrine cells in P21 flatmounts | 2812.4±162.8 pixels  (rel. µm^2^),  N=7 eyes  515 cells | 5854.9±811.2 pixels  (rel. µm^2^),  N=6 eyes 332 cells | 2.1-fold increase | p=0.002 |
| TH^+^ amacrine cells in P21 flatmounts | 1122.6±47.0 pixels  (rel. µm^2^),  N=9 eyes,  595 cells | 2686.2±251.5 pixels  (rel. µm^2^),  N=6 eyes  206 cells | 2.4-fold increase | p<0.0001 |
| SMI-32^+^ RGCs in P21 flatmounts | 2166.0±83.9 pixels  (rel. µm^2^),  N=3 eyes, 391 cells | 6626.5±892.2 pixels  (rel. µm^2^),  N=3 eyes, 253 cells | 3.1-fold increase | p=0.008 |
