# Supplementary material for: Cell-Type Specific Roles for PTEN in Establishing a Functional Retinal Architecture
Source: PLoS One. 2012 Mar 5;7(3):e32795. doi: 10.1371/journal.pone.0032795 (PMC3293905; doi:10.1371/journal.pone.0032795)
Supplement: Table S2 — Analysis of horizontal and amacrine cell spacing and wholemount cell counts in P21 wild-type and Pten cKO retinal flatmounts. (DOC) [file pone.0032795.s006.doc]

**Table S2. Analysis of horizontal and amacrine cell spacing and wholemount cell counts in P21 wild-type and *Pten* cKO retinal flatmounts.** n/s=not significant.

| **Voronoi Domain Regularity Index** | Wild-type | *Pten* cKO | fold-change  (wild-type/*Pten* cKO) | P value |
| --- | --- | --- | --- | --- |
| calbindin^+^  horizontal cells | 4.94 ± 0.13  N=4 eyes  4335 cells | 3.84±0.38  N=4 eyes  2224 cells | 1.3-fold lower  (77% less) | P=0.03 |
| TH^+^  amacrine cells | 2.83±0.08  N=8 eyes,  2315 cells | 1.93±0.10 N=5 eyes;  1026 cells | 1.5-fold lower  (67% less) | P<0.0001 |
| **Nearest Neighbor Regularity Index** |  |  |  |  |
| calbindin^+^  horizontal cells | 4.66 ± 0.13  N=4 eyes  4335 cells | 3.81±0.35  N=4 eyes  2224 cells | 1.2-fold lower  (83% less) | P=0.06  n/s |
| TH^+^  amacrine cells | 2.73±0.04  N=8 eyes,  2315 cells | 2.12±0.07 N=5 eyes;  1026 cells | 1.3-fold lower  (77% less) | P<0.0001 |
| **Wholemount Cell Counts** |  |  |  |  |
| calbindin^+^  horizontal cells | 10510 ±320.2 N=4 eyes | 7477 ± 178.2 N=4 eyes | 1.4-fold lower  (71% less) | P<0.0001 |
| TH^+^  amacrine cells | 367.1 ± 11.18 N=8 eyes | 229.7 ± 5.470 N=5 eyes | 1.6-fold lower  (63% less) | P<0.0001 |
