# Supplementary material for: Cell-Type Specific Roles for PTEN in Establishing a Functional Retinal Architecture
Source: PLoS One. 2012 Mar 5;7(3):e32795. doi: 10.1371/journal.pone.0032795 (PMC3293905; doi:10.1371/journal.pone.0032795)
Supplement: Table S3 — Analysis of electroretinogram responses in adult wild-type and Pten cKO mice. (DOC) [file pone.0032795.s007.doc]

**Table S3. Analysis of electroretinogram responses in adult wild-type and *Pten* cKO mice (n=7 wild-type, n=5 mutant mice).**

**Scotopic Intensity Response (SIR):**

| **Measure** | **From Figure** | | **Significant difference?** | **Stat values:** |
| --- | --- | --- | --- | --- |
| A-wave amplitude | S1A | | No | P = 0.819 |
| A-wave implicit time | S1B | | No | P = 0.245 |
| B-wave amplitude | S1C | | No | P = 0.650 |
| B-wave implicit time | S1D | | No | P = 0.330 |
| Oscillatory Potential isolation: | |  | | |
| Amplitude (wavelet) | 6D | | No | P = 0.545 |
| Frequency (wavelet) | 6E | | Main effect | rmANOVA: F_1,10_ = 7.775, P = 0.019 |
| Latency (wavelet) | 6F | | Main effect | rmANOVA: F_1,10_ = 8.386, P = 0.016 |

**Photopic Intensity Response (PIR):**

| **Measure** | **From Figure** | | **Significant difference?** | **Stat values:** |
| --- | --- | --- | --- | --- |
| B-wave amplitude | S1E | | No | P = 0.896 |
| B-wave implicit time | S1F | | No | P = 0.197 |
| Oscillatory Potential isolation: | |  | | |
| Amplitude (wavelet) | S2D | | No | P = 0.261 |
| Frequency (wavelet) | S2E | | Main effect | rmANOVA: F_1,9_ = 7.061, P = 0.026 |
| Latency (wavelet) | S2F | | No | P = 0.836 |

**Double Flash Response (DF):**

| **Measure** | **From Figure** | | **Significant difference?** | **Stat values:** |
| --- | --- | --- | --- | --- |
| B-wave amplitude | S1G | | Interaction (wt vs. ko) | rmANOVA: F_9,90_ = 3.059, P = 0.003 |
| B-wave implicit time | S1H | | No | P = 0.983 |
| Oscillatory Potential isolation: | |  | | |
| Amplitude (wavelet) | 6J | | Interaction (wt vs. ko) | rmANOVA: F_9,81_ = 2.919, P = 0.005 |
| Frequency (wavelet) | 6K | | Main effect | rmANOVA: F_1,8_ = 5.796, P = 0.043 |
| Latency (wavelet) | 6L | | No | P = 0.053 |
