# Supplementary material for: Cell-Type Specific Roles for PTEN in Establishing a Functional Retinal Architecture
Source: PLoS One. 2012 Mar 5;7(3):e32795. doi: 10.1371/journal.pone.0032795 (PMC3293905; doi:10.1371/journal.pone.0032795)
Supplement: Table S4 — Analysis of optokinetic contrast sensitivity responses in adult wild-type and Pten cKO mice. (DOC) [file pone.0032795.s008.doc]

**Table S4. Analysis of optokinetic contrast sensitivity responses in adult wild-type and *Pten* cKO mice (n=6 wild-type, n=6 mutant mice).**

| **From Figure** | **Significant difference?** | **Statistical test** | **Stat values:** |
| --- | --- | --- | --- |
| 8H | Yes (at 0.275 cycles/degree) | Mann-Whitney U-test | U=2.00, U’=22.00, P=0.034 |
| 8I | Yes | Linear Mixed Model (LMM) | F_1,25.012_ = 8.333,  P = 0.002 |
| Post-hoc tests: |  | | |
| 8I | Yes (wt vs. affected ko) | Least Significant Difference (LSD) | P < 0.001 |
| 8I | Yes (unaffected ko vs. affected ko) | Least Significant Difference (LSD) | P = 0.003 |
